# Blocking the Hormone Receptors Modulates NLRP3 in LPS-Primed Breast Cancer Cells

**DOI:** 10.3390/ijms24054846

**Published:** 2023-03-02

**Authors:** Shaimaa Hamza, Ekaterina E. Garanina, Mohammad Alsaadi, Svetlana F. Khaiboullina, Gulcin Tezcan

**Affiliations:** 1 Institute of Fundamental Medicine and Biology, Kazan Federal University, 420008 Kazan, Russia; 2 Department of Fundamental Sciences, Faculty of Dentistry, Bursa Uludag University, Bursa 16059, Turkey

**Keywords:** breast cancer, estrogen receptor alpha, tamoxifen, progesterone receptor, mifepristone, NLRP3

## Abstract

NOD-like receptor protein 3 (NLRP3) may contribute to the growth and propagation of breast cancer (BC). The effect of estrogen receptor-α (ER-α), progesterone receptor (PR), and human epidermal growth factor receptor 2 (HER2) on NLRP3 activation in BC remains unknown. Additionally, our knowledge of the effect of blocking these receptors on NLRP3 expression is limited. We used GEPIA, UALCAN, and the Human Protein Atlas for transcriptomic profiling of NLRP3 in BC. Lipopolysaccharide (LPS) and adenosine 5′-triphosphate (ATP) were used to activate NLRP3 in luminal A MCF-7 and in TNBC MDA-MB-231 and HCC1806 cells. Tamoxifen (Tx), mifepristone (mife), and trastuzumab (Tmab) were used to block ER-α, PR, and HER2, respectively, on inflammasome activation in LPS-primed MCF7 cells. The transcript level of *NLRP3* was correlated with ER-ɑ encoding gene *ESR1* in luminal A (ER-α^+^, PR^+^) and TNBC tumors. NLRP3 protein expression was higher in untreated and LPS/ATP-treated MDA-MB-231 cells than in MCF7 cells. LPS/ATP-mediated NLRP3 activation reduced cell proliferation and recovery of wound healing in both BC cell lines. LPS/ATP treatment prevented spheroid formation in MDA-MB-231 cells but did not affect MCF7. HGF, IL-3, IL-8, M-CSF, MCP-1, and SCGF-b cytokines were secreted in both MDA-MB-231 and MCF7 cells in response to LPS/ATP treatment. Tx (ER-α inhibition) promoted NLRP3 activation and increased migration and sphere formation after LPS treatment of MCF7 cells. Tx-mediated activation of NLRP3 was associated with increased secretion of IL-8 and SCGF-b compared to LPS-only-treated MCF7 cells. In contrast, Tmab (Her2 inhibition) had a limited effect on NLRP3 activation in LPS-treated MCF7 cells. Mife (PR inhibition) opposed NLRP3 activation in LPS-primed MCF7 cells. We have found that Tx increased the expression of NLRP3 in LPS-primed MCF7. These data suggest a link between blocking ER-α and activation of NLRP3, which was associated with increased aggressiveness of the ER-α^+^ BC cells.

## 1. Introduction

NOD-like receptor protein 3 (NLRP3), an intracellular oligomeric protein complex, is formed followed by the priming of pathogen-associated molecular patterns (PAMPs) and damage-associated molecular patterns (DAMPs) [1,2]. A secondary signal, such as adenosine 5′-triphosphate (ATP), is required to trigger the polymerization and formation of an active NLRP3 complex [3,4]. There is considerable evidence suggesting the role of NLRP3 in tumor pathogenesis, although results are often controversial. Some studies have shown that NLRP3 accelerates tumor progression by inducing epithelial-mesenchymal transition (EMT) and angiogenesis [5,6]. Others have revealed limited tumor cell survival due to pyroptotic cell death [7,8]. The mechanisms of these conflicting data remain largely unknown.

Previously, we demonstrated that the degree of NLRP3 inflammasome activation varies among cancer cell lines, resulting in different cancer cell growth patterns [9]. We also have shown that a high level of NLRP3 expression in cancer cells promotes more aggressive tumor features, such as enhanced proliferation and migration, compared to cancer cells that express a lower level of NLRP3 [9]. In addition, overexpression of NLRP3 enhanced resistance to chemotherapy [10,11]. Previous studies demonstrated that these anticancer therapeutics link chemoresistance to promoting NLRP3 expression [11,12]. Therefore, predicting the effects of chemotherapy drugs on tumor growth requires prior testing of the NLRP3 expression status.

Hormone-based therapeutics are often used to treat breast and ovarian cancers [13,14,15]. Tamoxifen (Tx), an estrogen receptor alpha (ER-ɑ) antagonist, is recommended as adjuvant therapy in breast cancer (BC) patients with an ER-ɑ expressing, lymph-node-negative or lymph-node-positive tumor [16,17]. Additionally, a progesterone receptor (PR) antagonist, mifepristone (mife), has been shown to inhibit the growth of ovarian cancer and BC cells with high PR expression [18,19]. Monoclonal-antibody-based immunotherapeutics, such as trastuzumab (Tmab), that target human epidermal growth factor receptor 2 (HER2) are used to treat HER2-expressing BC cancer cells [20,21,22,23]. Our understanding of the therapeutic mechanisms of these drugs is based on the inhibition of hormone-activated intracellular pathways [16,17,18,19,24]. However, there is a limited understanding of the interaction between NLRP3 activation and hormone receptor inhibition in cancer cells, which could contribute to the therapeutic efficacy of these drugs.

BC tumors are classified based on the expression of receptors ER-α and PR as well as HER2 [25]. Tumors lacking ER-α/PR and HER2 are classified as triple-negative BC (TNBC) [26]. The majority, 70%, of BC tumors express hormone receptors ER-α and/or PR [27], whereas 20–30% have amplification or overexpression of HER2 [28]. The TNBCs constitute the smaller fraction, 12–17%, of BC tumors [29]. These receptors’ role in BC’s pathogenesis is established; however, our understanding of the ER-α, PR, and HER2 expression on NLRP3 inflammasome is limited. The tumor-specific variations in these receptors’ expression could contribute to mechanisms of inflammasome-associated tumor growth. This study aims to address this knowledge gap by investigating the NLRP3 expression effect on BC proliferation in ER-α, PR, and HER2 expressing cells, as well as in TNBC.

The effect of NLRP3 activation on cancer progression in TNBC, MDA-MB-231 and HCC1806 cells, and MCF7 cells, expressing ER-α, PR, and low to moderate levels of HER2, was investigated. ER-α antagonist Tx [30], PR antagonist mife [31] and Tmab inhibiting HER2 [32] were used to analyze the effect of these receptors’ expression on NLRP3 expression in MCF7 cells. We found that TNBC cells MDA-MB-231 and HCC1806 have a higher NLRP3 expression than hormone-receptor-expressing BC cell MCF7. NLRP3 activation increased proliferation, migration, and release of inflammatory cytokines in TNBC cell MDA-MB-231. In contrast, MCF7 cells expressing these receptors had low NLRP3 activation associated with reduced BC cell growth, migration, and lower production of proinflammatory cytokines. In LPS-treated cells, Tx induced NLRP3 expression and caused a more aggressive MCF7 cell phenotype similar to that when cells were treated with LPS/ATP. In contrast, blocking PR with mife reduced NLRP3 activation in LPS/ATP-primed MCF7 cells. Blocking HER2 by Tmab had a limited effect on NLRP3 expression in MCF7 cells.

## 2. Results

### 2.1. Transcriptional Levels of NLRP3 in BC Tumors and Cell Lines

**Figure 1:**
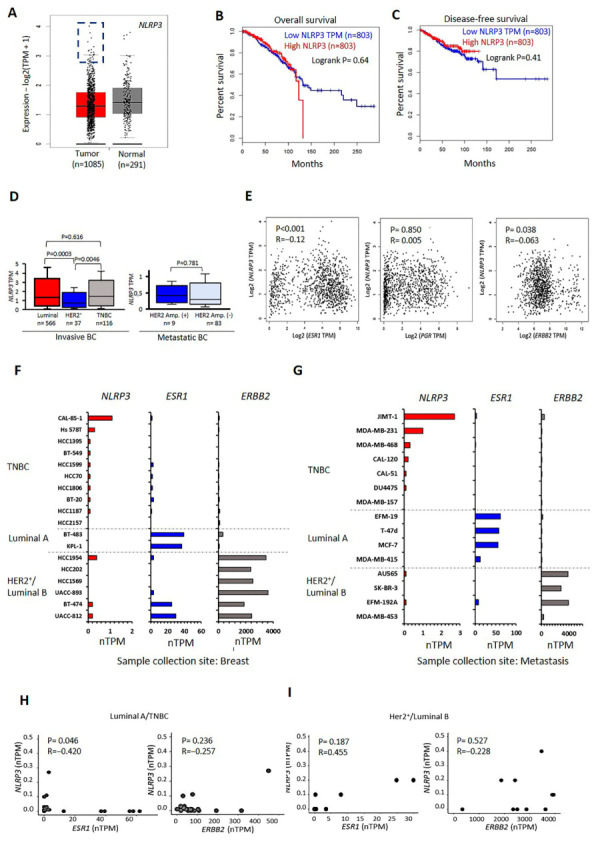
The expression of NLRP3 in BC tumors and the representative BC cell type-specific expressed NLRP3 transcripts. (**A**) The comparison of NLRP3 transcript levels between BC tumors and normal tissues (GEPIA; n = 1376). (**B**) NLRP3 dependent overall and (**C**) disease-free survival of BC patients (GEPIA, n = 1606). (**D**) NLRP3 expression in major subclasses of BC (UALCAN; n = 811). (**E**) The correlation between NLRP3, ESR1, PGR, and ERBB2 in BC tumors (GEPIA, n = 1067). (**F**) The transcript levels NLRP3, ESR1, PGR, and ERBB2 in BC cells from the primary tumor site and (**G**) the metastasis site (HPA). Cells were manually selected from HPA. Cell lines with BRCA1 mutations or controversial molecular classifications were excluded [34,35]. (**H**) The correlation between NLRP3 and ESR1 and ERBB2 in luminal A and TNBC cell lines and (**I**) HER2^+^ and luminal B cell lines. Luminal A: ER-α^+^, PR^+^; luminal B: ER-α^+^, HER2^+^; TNBC: triple-negative. TPM: transcripts per million; nTPM: normalized transcripts per million.

The mean RNA transcript level of the NLRP3 was lower in BC tumor samples than in normal tissues in the Cancer Genome Atlas (TCGA) and Genotype-Tissue Expression (GTEx)-derived BC patients (Figure 1A). However, BC patients with high NLRP3 expressed tumors had a lower overall and disease-free survival (Figure 1B,C). BC tumors expressing only ER-α or ER-α and PR were classified as luminal A; those expressing ER-α and HER2 were classified as luminal B; and those not expressing ER-α, PR, or HER2 were classified as triple-negative [33]. The transcript level of NLRP3 was detected as higher in the luminal and triple-negative groups (Figure 1D). Accordingly, we analyzed the correlation of the RNA transcript levels between ER-α encoding gene ESR1, PR encoding gene PGR, and HER2 encoding gene ERBB2 in tumor samples of BC patients. The results indicated that the RNA transcript level of NLRP3 was negatively correlated with ESR1 (*p* < 0.001) and ERBB2 (*p* = 0.038). In contrast, no correlation was determined between NLRP3 and PGR (Figure 1E). Depending on the molecular classification of BC, the RNA transcript level of NLRP3 was higher in TNBC cells compared to luminal A group in BC cells derived from the primary tumor site (Figure 1F) and metastatic site (Figure 1G). According to the Human Protein Atlas (HPA) database, a negative correlation was determined between the RNA transcript level of NLRP3 and ESR1 in luminal A (ER-α^+^, PR^+^) and TNBC cells (*p* = 0.046). In contrast, no correlation between NLRP3 and ESR1 was detected in luminal B (ER-α^+^ and HER2^+^) and HER2^+^ BC cell lines (Figure 1H,I). No correlation was detected between NLRP3 and ERBB2 in luminal A, TNBC, luminal B, and HER2^+^ BC cell lines. These data suggest that ER-α coding ESR1 gene expression could inhibit RNA expression of NLRP3 in luminal A cells. In contrast, the absence of ESR1 transcripts liberates NLRP3 expression in TNBC cells. However, NLRP3 expression could not be affected by ESR1 in luminal type B cells.

### 2.2. NLRP3 Protein Levels Vary in TNBC and Luminal A Type Cells

**Figure 2:**
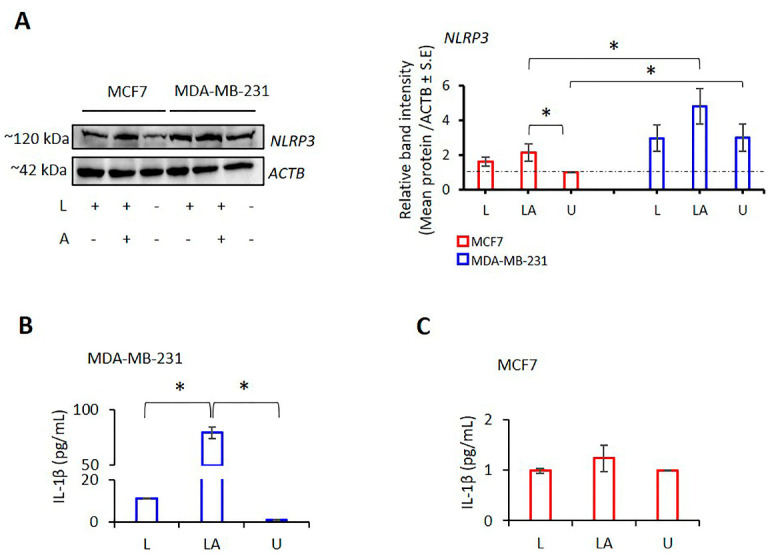
NLRP3 expression and IL-1β secretion levels in MDA-MB-231 and MCF-7 cells. LPS (1 µg/mL, 3 h) was used for NLRP3 induction, followed by ATP (5 mM ATP; 25 min) inflammasome formation in MDA-MB-231 and MCF-7 cells. (**A**) Western blot analysis of NLRP3 protein expression in MDA-MB-231 and MCF7 cells; (**B**) ELISA assessment of IL-1β secretion in MDA-MB-231 cells; (**C**) ELISA detection of IL-1β release in MCF7 cells. Data represent three technical repeats. L: LPS only, LA: LPS/ATP, U: untreated. *p* value was calculated using the one-way ANOVA model with Tukey’s post hoc tests. * *p* < 0.05.

LPS/ATP increased NLRP3 protein expression in TNBC MDA-MB-231, HCC1806, and luminal A MCF7 cells (Figure 2A; Figure S1). However, untreated MDA-MB-231 cells produced more NLRP3 protein compared to untreated MCF7 cells, which resulted in a higher concentration of NLRP3 production of LPS/ATP-treated MDA-MB-231 cells compared to LPS/ATP-treated MCF7 cells (Figure 2A). In addition, although the NLRP3 expression of untreated and LPS/ATP-treated HCC1806 cells was lower than MDA-MB-231 cells, it was higher than in MCF7 cells (Figure S1, Table S1). The secretion of interleukin (IL)-1β was increased in LPS/ATP-treated MDA-MB-231 cells (*p* < 0.001) compared to the control. As anticipated, this cytokine production was less affected in MCF-7 cells than in the control (Figure 2B,C, Table S2). These data demonstrate that LPS/ATP activates NLRP3 in MDA-MB-231 cells, which are TNBC. However, inflammasome induction had a limited effect on MCF-7, expressing ER-α^+^, PR^+^, and low-Her2^+^ cells.

### 2.3. The Proliferation of MDA-MB-231 and MCF-7 Cells Differ after NLRP3 Activation

**Figure 3:**
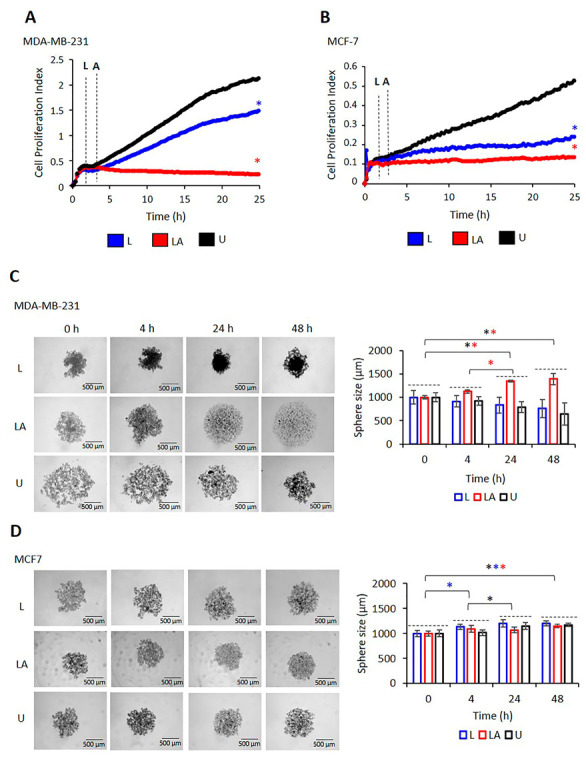
The effect of NLRP3 activation on proliferation and sphere formation of MDA-MB-231 and MCF-7 cells. Cells were incubated with LPS (1 µg/mL, 3 h) to activate NLRP3, followed by ATP treatment (5 mM ATP; 25 min) to form an inflammasome complex. Images were captured before LPS treatment (time 0), 30 min after ATP treatment (time 4), and 24–48 h after LPS/ATP treatment. The effect of LPS only and LPS/ATP on proliferation kinetics of MDA-MB-231 (**A**) and MCF-7 cells (**B**). Data represent three technical repeats. *p* value was calculated at the 48th hour using the one-way ANOVA and Tukey test. The sphere formation capacity of MDA-MB-231 (**C**) and MCF-7 cells (**D**) after NLRP3 activation. Images were analyzed using Image J software. *p* value was calculated using the one-way ANOVA and Tukey test. Data represent five technical repeats. * Comparison of untreated, LPS-only, and LPS-ATP-treated cells at different incubation times. ◊: Comparison of LPS-only and LPS-ATP-treated cells to untreated cells at the 48 h time point. The mean difference is significant at 0.05 for all analyses (black: untreated cells, blue: LPS-only-treated cells, red: LPS/ATP-treated cells). U: untreated, L: LPS, LA: LPS/ATP.

MDA-MB-231 and MCF-7 cells treated with LPS only and LPS/ATP were used to investigate the real-time impact of NLRP3 activation on cell growth. Cell proliferation rate kinetics in untreated, LPS-only, and LPS/ATP-treated MDA-MB-231 cells were higher than in MCF7 cells (Figure 3A,B). LPS/ATP substantially attenuated cell growth in MDA-MB-231 and MCF-7 cells compared to untreated and LPS-only-treated cells (Figure 3A,B).

The effect of LPS/ATP on the tumor cell sphere differed in MDA-MB-231 and MCF-7 cells. In MDA-MB-231 (TNBC; ER-α^−^, PR^−^, Her2^−^), untreated and LPS-only-treated MDA-MB-231 cells started forming spheres in the first 4 h, forming a whole sphere from large-size diffuse aggregates in 24 h, which resulted in a smaller size of the spheres. After 24 and 48 h, there was no difference in the size of spheres formed by untreated and LPS-only-treated MDA-MB-231 cells. However, although LPS/ATP-treated MDA-MB-231 cells started to form spheres in 4 h, the size of the spheres increased during 24 h (*p* = 0.008, Figure 3C, Table S3), and then did not change until the 48 h time point. At 48 h, the size of the LPS-ATP-treated MDA-MB-231 spheres was larger than the untreated spheres (*p* = 0.048, Table S4). In contrast, the size of untreated MCF7 cells was unchanged for the first 4 h. However, they became enlarged by the 24 h time point (*p* = 0.020; Figure 3D, Table S3). The size of the spheres remained the same for the remaining incubation time. Interestingly, treatment with LPS only induced rapid growth of the sphere in the first 4 h (*p* = 0.023, Figure 3D, Table S3). However, the size of these spheres remained unchanged for the remaining time. LPS/ATP did not affect sphere sizes compared to untreated MCF7 cells. Our data suggest that NLRP3 activation was more pronounced in MDA-MB-231, a TNBC cell, and it was also characterized by sphere growth. In contrast, limited capacity to activate NLRP3 in ER-α^+^, PR^+^, and HER-2^low+^ MCF7 cells was associated with a lack of effect on sphere formation and growth.

**Figure 4:**
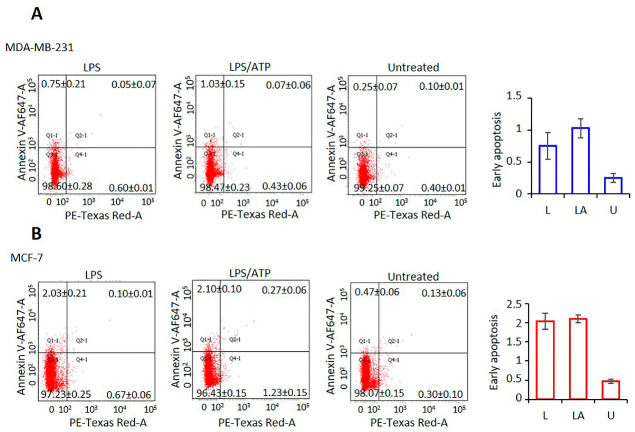
The effect of NLRP3 activation on cell death in MDA-MB-231 and MCF-7 cells. Cells were incubated with LPS (1 µg/mL, 3 h) to activate NLRP3 followed by ATP treatment (5 mM ATP; 25 min) to form an inflammasome complex. Images were analyzed using FlowJo software. (**A**) MDA-MB-231 cells; (**B**) MCF-7 cells. L: LPS only, LA: LPS/ATP, U: untreated. *p* value was calculated using the one-way ANOVA model with Tukey’s post hoc tests. Data represent three technical repeats.

LPS only and LPS/ATP did not affect the rate of early apoptosis (annexin V (+)/PI (−)) in MDA-MB231 and MCF7 (Figure 4A,B, Table S5). These data suggest that NLRP3 activation failed to induce apoptotic cell death independent of ER-α^+^, PR^+^, and Her2^+^ expression status in BC cells.

### 2.4. NLRP3 Expression Reduces the Migration Capacity of BC Cells

**Figure 5:**
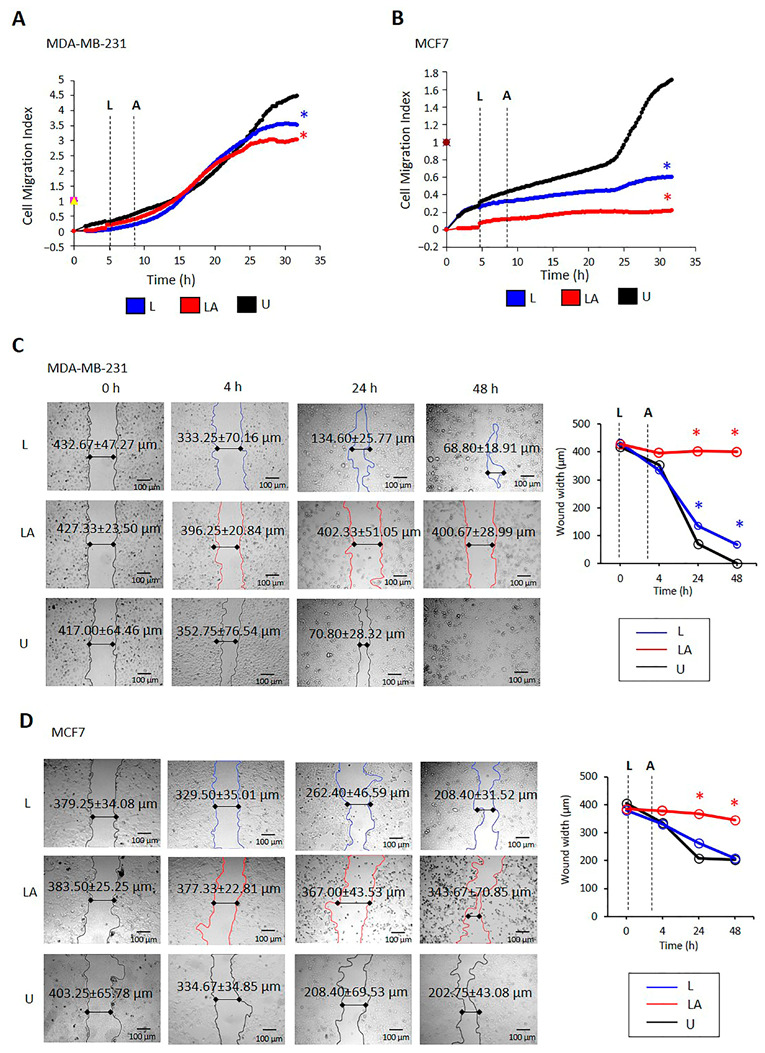
The effect of NLRP3 activation on the migration of MDA-MB-231 and MCF-7 cells. Cells were incubated with LPS (1 µg/mL, 3 h) to activate NLRP3, followed by ATP treatment (5 mM ATP; 25 min) to form an inflammasome complex. Images were captured before LPS treatment (time 0), 30 min after ATP treatment (time 4), and 24–48 h after LPS/ATP treatment. The effect of LPS only and LPS/ATP on migration kinetics in MDA-MB-231 (**A**) and MCF-7 (**B**) cells. Light-colored cells are attached, whereas dark color cells are detached [36]. Data represent three technical repeats. *p* value was calculated at the 24th hour using the one-way ANOVA and Tukey test. The effect of LPS only and LPS/ATP on the wound area size in MDA-MB-231 (**C**) and MCF-7 (**D**) cells. Images were analyzed using Image J software. The *p* value was calculated using the one-way ANOVA and Tukey test. Data represent five technical repeats. * The mean difference is significant at 0.05 for all analyses (blue: LPS-only-treated cells, red: LPS/ATP-treated cells). U: untreated, L: LPS, LA: LPS/ATP.

The effect of NLRP3 activation on the migration of MDA-MB-231 and MCF-7 cells was investigated using uncoated CIM-16 xCELLigence plates. The untreated MDA-MB-231 cell migration rate through uncoated membranes was higher than MCF7 cells (Figure 5A,B). The migration rate decreased following LPS only and LPS/ATP treatment compared with untreated cells in both cell lines (Figure 5A,B).

Likewise, in the scratch wound healing assay, the wound closing rate in untreated MDA-MB-231 cells was higher than that of MCF7 cells in 48 h (t = 3.26; *p* = 0.026, Figure 5C,D). In untreated MDA-MB-231 cells, the wounded area closed within 48 h (*p* < 0.001, Figure 5C, Table S6), whereas it remained open in MCF7 cells. LPS only reduced wound healing in 24 h in MDA-MB-231 compared to untreated cells (*p* = 0.027, Figure 5C, Table S7). In contrast, LPS only did not affect the wound closing rate in MCF-7 compared to untreated cells in 24 or 48 h. LPS/ATP reduced the speed of wound healing over 48 h in MDA-MB231 and MCF7 cells compared to untreated and LPS-only-treated cells (*p* < 0.001). However, most spreading MDA-MB-231 cells were attached, motile cells, whereas MCF7 cells were detached and immotile [36] (Figure 5C,D, Table S7).

### 2.5. Effect of NLRP3 Activation on Cytokine Release in BC Cells

**Figure 6:**
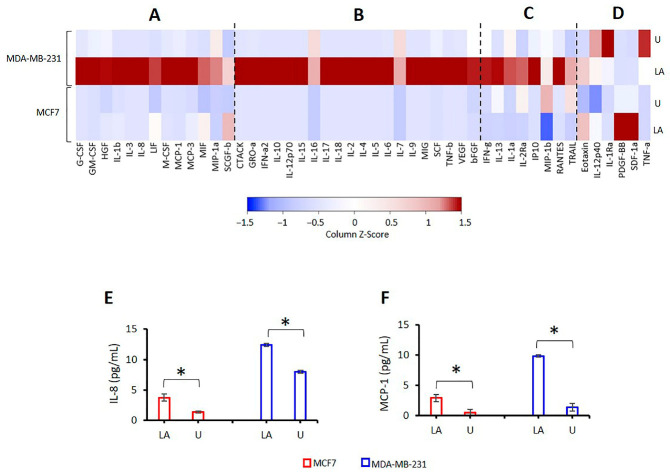
The heat map analysis of cytokine secretion in MDA-MB-231 and MCF-7 cells after NLRP3 activation. After inflammasome activation, cytokine secretion in MDA-MB 231 and MCF7 cells was analyzed using Bio-Plex Pro Cytokine Screening Panel. Results were calculated with BioPlex Manager v.6.1. A minimum of 50 beads per well for each cytokine were analyzed. (**A**) Cytokines released by MDA-MB-321 and MCF7 cells; (**B**) cytokines activated only in MDA-MB-231; (**C**) cytokines activated in MDA-MB-231 but decreased in MCF7; (**D**) cytokines decreased in MDA-MB-231 but unaffected in MCF7. (**E**) ELISA assessment of IL-8 secretion in MCF7 and MDA-MB-231 cells; (**F**) ELISA detection of MCP-1 release in MCF7 and MDA-MB-231 cells. *p* value was calculated using the Mann–Whitney U test for (**A–D**) and using an independent sample t test for (**E**,**F**). * The mean difference is significant at 0.05. U: untreated, LA: LPS/ATP. Data represent three technical repeats.

The level of 48 cytokines was measured 24 h after treatment of BC cells with LPS/ATP. Secretion levels of granulocyte colony-stimulating factor (G-CSF), IL-18, interferon gamma-induced protein 10 (IP10), monocyte chemoattractant protein 1 (MCP-1), macrophage inflammatory protein-1 alpha (MIP-1a), and stem cell factor (SCF) were significantly higher in untreated MDA-MB-231 cells compared to untreated MCF7 cells (Figure 6A–F; Tables S8 and S9). These data suggest the higher proinflammatory capacity of MDA-MB-231 cells compared to MCF7 cells.

LPS/ATP treatment induced the secretion of proinflammatory HGF, IL-3, IL-8, M-CSF, MCP-1, and SCGF-b in MDA-MB-231 and MCF7 cells (Figure 6A, Tables S10 and S11). However, changes in these cytokines’ levels were significant only in MDA-MB-231 cells. Secretion of cutaneous T-cell-attracting chemokine (CTACK), SCF, IL-1a, and regulated upon activation, normal T cell expressed and presumably secreted (RANTES) was increased in LPS/ATP-treated MDA-MB-231 cells (Figure 6B,C, Tables S10 and S11). In contrast, LPS/ATP did not affect the secretion of these cytokines in MCF7 cells (Figure 6B,C, Tables S10 and S11). These data indicate that inflammasome activation induced a proinflammatory cytokine secretion in TNBC MDA-MB-231 BC cells. These cells are characterized by high NLRP3 activation capacity. In contrast, MCF7 cells expressing hormone receptors had modest cytokine secretion upon inflammasome activation. It should be noted that MCF7 cells have a limited capacity for inflammasome activation [9].

### 2.6. Targeting Hormone Receptors affect NLRP3 Status in MCF7 Cells

**Figure 7:**
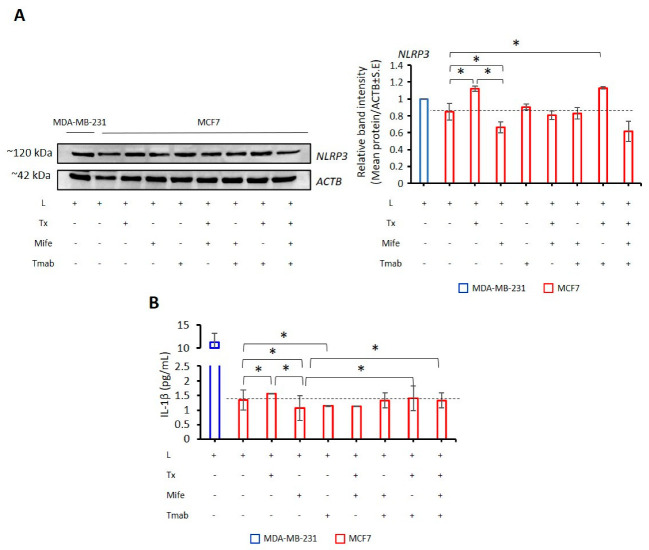
Effect of Tx, mife, and Tmab on NLRP3 expression in MCF-7 cells. LPS (1 µg/mL; 3 h) was used to prime NLRP3 transcription and translation in MCF-7 cells. Tx (5 µM; 24 h), mife (10 nM; 48 h), and Tmab (10 μg/mL; 48 h) were added 3 h after LPS treatment. For positive control of NLRP3 inflammasome activation, MDA-MB-231 cells were primed LPS. (**A**) Western blot analysis of NLRP3 protein expression in MCF7 cells. Data represent two technical repeats. (**B**) ELISA detection of IL-1β secretion in MCF7 cells. Data represent technical repeats. *p* value was calculated using the one-way ANOVA model with Tukey’s post hoc tests for all analyses. * *p* < 0.05; L: LPS, Tx: tamoxifen, mife: mifepristone, Tmab: trastuzumab.

LPS primed MCF7 (ER-α+, PR+, HER-2^low+^) cells treated with Tx, mife, and Tmab to block ER-α, PR, and HER2, respectively. In LPS-primed MCF7 cells, blocking ER-α with Tx induced NLRP3 expression (Figure 7A, Table S12). In contrast, mife, which targets PR, reduced NLRP3 expression compared to LPS-only MCF7 cells. Additionally, LPS/Tx increased the secretion of IL-1β (*p* = 0.05; Figure 7B), whereas LPS/mife decreased the release of this cytokine compared to LPS-only-treated MCF7 cells (*p* = 0.010, Figure 7B, Table S13).

Tmab, which targets HER2, did not affect NLRP3 protein expression and IL-1β secretion (Figure 7A,B). Upon treatment with LPS/Tx/mife and LPS/Tx/mife/Tmab, expression of NLRP3 was reduced compared to LPS only. Also, the secretion of IL-1β was lower than LPS only (Figure 7A,B). In contrast, LPS/Tx/Tmab had a limited effect on NLRP3 expression and IL-1β secretion, which remained similar to that in cells treated with LPS/Tx (Figure 7A,B). These findings suggest that the effects of ER-α and PR antagonists on NLRP3 expression vary. While Tx blocking ER-α induces inflammasome expression, blocking PR with mife reduced NLRP3 expression in LPS-primed MCF7 cells.

### 2.7. NLRP3 Activation Combined with Tx Blocking ER-α Promotes MCF7 Cells Migration and Proliferation

**Figure 8:**
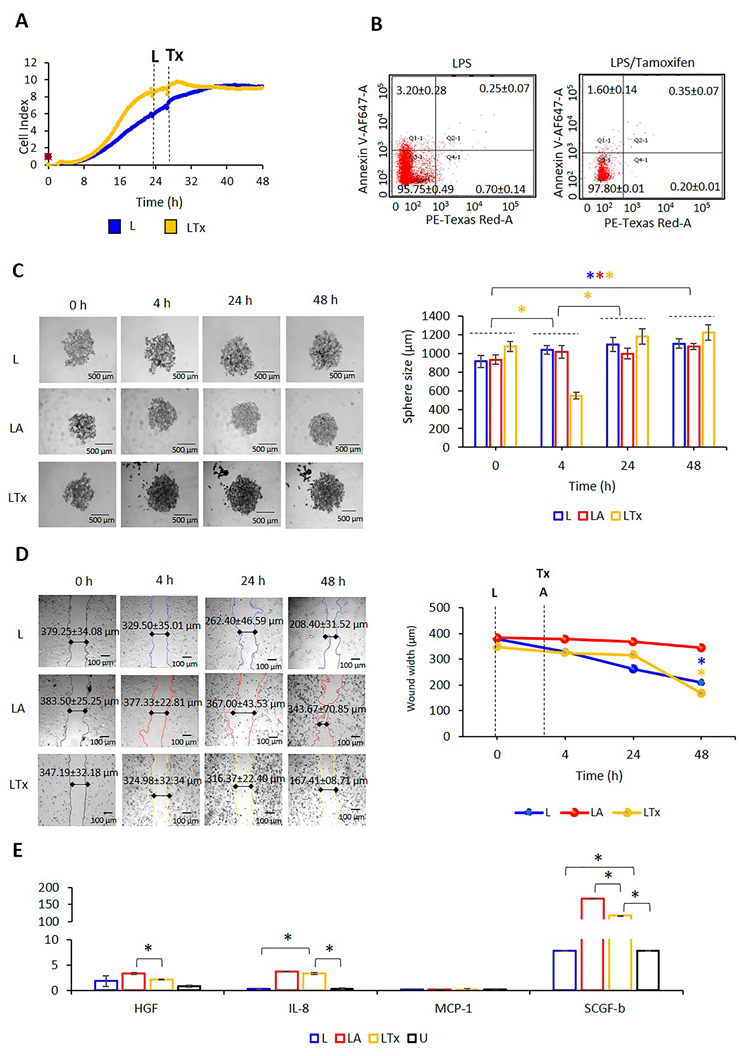
Tx-mediated ER-α suppression affects cell proliferation, sphere formation, and wound healing in LPS/ATP-treated MCF7 cells. (**A**) The effect of L-Tx on proliferation kinetics; (**B**) apoptosis; (**C**) the sphere formation; (**D**) the in vitro closure of the wound healing area; (**E**) HGF, IL-8, MCP-1, and SCGF-b secretion. Data represent three technical repeats for (**A**,**B**,**E**) and five technical repeats for (**C**,**D**). The *p*-value was calculated using the one-way ANOVA model with Tukey’s post hoc tests for (**A**–**C**) and independent-samples Kruskal-Wallis test for (**E**). * *p* < 0.05 (blue: LPS-only-treated cells, red: LPS/ATP-treated cells, yellow: LPS/Tx-treated cells). L: LPS, LA: LPS/ATP, LTx: LPS/tamoxifen, U: untreated.

It was only blocking ER-α with Tx increased NLRP3 expression. Therefore, the effect of Tx on cell proliferation, migration, and sphere formation was analyzed in MCF7 cells. MCF-7 cells treated with LPS only or LPS/Tx to investigate the real-time impact of NLRP3 activation on cell growth. Cell proliferation kinetics indicated that LPS/Tx had a limited effect on the proliferation rate of MCF-7 cells compared to LPS only (Figure 8A). Supporting these data, LPS/Tx did not affect the apoptotic cell counts compared to LPS only (Figure 8B). In addition, LPS/Tx reduced the sphere size at the initial 4 h (*p* < 0.001, Figure 8C, Table S14). At the later time points (48 h of incubation), the sphere sizes were significantly larger than LPS-only-treated cells (*p* = 0.014, Figure 8C, Table S15). These data suggest that when NLRP3 is activated, Tx-mediated suppression of ER-α could increase cell proliferation and enhance the sphere size.

A wound healing assay was used to investigate the effect of Tx suppression of ER-α on the migratory properties of MCF-7 cells after inflammasome activation. LPS/Tx increased the wound healing rate over 48 h (*p* < 0.001, Figure 8D, Table S16). The wound healing was higher than in LPS-only and LPS/ATP-treated MCF7 cells during 48 h (*p* < 0.001; Figure 8D; Table S17). These data suggest that Tx increased the migration ability of MCF7 cells when the inflammasome is activated. Supporting this, LPS/Tx enhanced the secretion of the inflammatory cytokines IL-8 and SCGF-b. These cytokines could promote cancer cell migration, invasion, and induction of BC stem-like cell (BSC) phenotype [37,38], compared to untreated and LPS-only-treated MCF cells (Figure 8E; Table S18). Interestingly, the secretion of MCP-1 was not affected by LPS/Tx treatment compared to LPS only. These data indicate that a combination of activated inflammasome and ER-α blockade with Tx in MCF7 cells could promote a more aggressive phenotype.

## 3. Discussion

NLRP3 expression was detected in high levels in many types of tumors, such as head and neck squamous cell carcinoma [39], laryngeal squamous cell carcinoma [40], bladder cancer [41], and prostate cancer [42], compared to their nontumor tissue samples. In addition, a study showed an increased level of NLRP3 in BC cell lines MDA-MB-231, MCF-7, and SKBR3 compared to normal mammary epithelial cells [43]. NLRP3 expression and IL-1β secretion of BC tumors promote myeloid-derived suppressor cell (MDSCs) and tumor-associated macrophage (TAM) infiltration to the tumor microenvironment and facilitate an inflammatory microenvironment for cancer progression [44]. Supporting this, in this study, high NLRP3 expression decreased overall and disease-free survival in TCGA- and GTEx-derived BC patients. However, except for luminal and TNBC, NLRP3 transcription level in BC tumors was lower than that of normal tissue. In addition, the NLRP3 transcript levels of luminal A and TNBC cells were inversely correlated with the transcription level of ER-α encoding gene ESR1. Therefore, our study evaluated the effect of NLRP3 on BC cells in a luminal A type cell line, MCF7, and TNBC type cell lines MDA-MB-231 and HCC1800, which differ in terms of ESR1 transcript level.

NLRP3 activation requires priming with PAMPs and a second stimulus for inflammasome oligomerization [45]. The oligomerized inflammasome targets pro-caspase-1 and releases the active enzyme [46]. The functional caspase-1 cleaves pro-IL-1β, liberating active cytokines [46]. In this study, we used LPS, a PAMPs ligand for toll-like receptor 4 (TLR4), to prime inflammasome, which induces the transcription of structural components of NLRP3 and pro-IL-1β [47,48]. ATP was the second stimulus to initiate the functional inflammasome assembly [49]. We have found the highest NLRP3 activation in MDA-MB-231, TNBC cell line (ER-α^−^, PR^−^, HER2^−^), among all investigated cell lines after LPS/ATP induction. In addition, the expression of NLRP3 was higher in HCC1806, TNBC cell line (ER-α^−^, PR^−^, HER2^−^), compared to MCF-7 (ER-α^+^, PR^+^, HER2^low+^) cells after LPS/ATP induction. Additionally, LPS/ATP-treated MDA-MB-231 cells secreted more IL-1β, which confirms NLRP3 activation [50]. In contrast, LPS/ATP failed to increase the secretion of this cytokine in MCF7 cells, which corroborates the lack of inflammasome activation in these cells. MDA-MB-231 and HCC1806 cells are TNBC [34,35,51]. In contrast, MCF7 cells express a high level of ER-α and PR and a basal level of HER2 [52]. Additional molecular characteristics differ between these cell lines. These differences are mutations in P53, KRAS2, BRAF, and NF1 genes present in MDA-MB-231, but they are absent in MCF7 and HCC1806 cells. In contrast, MCF7 has mutations in PIK3CA and GATA3 that are not found in MDA-MB-231 [53,54,55]. Recent studies have identified that RAS mutational activation can stimulate NLRP3 inflammasome [56,57]. Therefore, the presence of RAS pathway mutations could explain the highest expression of NLRP3 in MDA-MB-231 compared to MCF7 and HCC1806 cells. Interestingly, we found the higher NLRP3 expression in HCC1806 compared to MCF7 cells. In this study, in addition to RAS pathway mutations, the expression status of ER and PR could also affect NLRP3. Supporting our findings, other studies showed an inverse correlation between the expression of ER-α and tumor secretion of the NLRP3 product IL-1β in BC tumor tissues [58,59]. Therefore, these data suggest that TNBC cells would have a higher capacity to establish NLRP3-mediated inflammation when exposed to PAMPs.

Similar to our previous findings [9], it appears that proliferation is independent of the NLRP3 activation capacity of tumor cells. LPS/ATP treatment decreased proliferation in both MDA-MB-231 and MCF7. However, the size of the tumor spheres formed by MDA-MB-231 and MCF7 cells differed. LPS/ATP increased the size of the tumor spheres in MDA-MB-231 cells, which had a high NLRP3 expression and IL-1β release. In contrast, LPS/ATP in MCF7 cells did not affect the tumor sphere size, where NLRP3 and IL-β were low. These data corroborate our previous report demonstrating tumor sphere growth in PC3 and U138MG expressing a high level of NLRP3, while tumor sphere size was reduced in SH-SY5Y, A549, and MCF7, where the NLRP3 expression was low [9]. Differences in BSC counts in MDA-MB-231 and MCF7 could explain the sphere formation. It was demonstrated that MDA-MB-231 cells have a large BSC population, expressing CD44^high^ and CD24^low^ [60], whereas lower BSC counts were reported in MCF7 [61]. SCGF-b is a hypoxia-inducible cytokine [62] that maintains the stem-like cell characteristic in tumors [63,64,65]. We have found that inflammasome activation of MDA-MB-231 increased SCGF-b secretion compared to MCF7 cells. Additionally, the inflammasome-associated production of IL-1β was shown to stimulate the hypoxic signaling pathways [66,67]. Hypoxia triggers the self-renewal of stem-like cells and facilitates the growth of tumor spheres, representing an in vivo equivalent of malignant tumor progression [68,69]. Therefore, the large size of tumor spheres in LPS/ATP-treated MDA-MB-231 cells could be explained by hypoxia, indicated by the high SCGF-b and IL-1β secretion level. This hypoxia could result in BSC renewal, leading to the increased size of spheres.

One of the hallmarks of cancer progression is metastasis, indicating cancer cell migration and invasion of the adjacent tissues [70]. We have found that inflammasome activation is associated with higher migration of MDA-MB-231 compared to MCF7 cells. Our previous study showed that, after NLRP3 activation, cancer cells with a low NLRP3 expression capacity could reduce cell growth and migratory features. In contrast, highNLRP3-expressed cancer cells can survive after a rapid decrease in cell proliferation [9]. The secretion of VEGF and MMP13 was reduced after NLRP3 activation in MCF7 cells [9], indicating that NLRP3 activation leads to decreased migratory properties of MCF7 cells [71,72]. In contrast, in high NLRP3-expressed cancer cells, such as prostate cancer cell line PC3, the secretion of VEGF and MMP-13 was higher compared to MCF-7 cells [9]. In line with this, in this study, although LPS-ATP decreased the cell proliferation rate of both MCF7 and MDA-MB-231 cells, the migration rate of MCF7 cells decreased, and MDA-MB-231 cells could migrate similarly to untreated cells. Supporting this, we observed motile cell spreading of MDA-MB-231 cells, while the detached MCF7 cells were immotile. In addition, inflammasome activation induced the secretion of HGF, IL-3, IL-8, M-CSF, and MCP-1 in both MDA-MB-231 and MCF7 cells. However, the level of secreted cytokines was higher in MDA-MB-231 compared to MCF7 cells. HGF, IL-8, and MCP-1 were shown to facilitate cancer cell migration, invasion, and metastasis by promoting EMT [73,74,75,76,77]. TLR4-mediated inflammasome activation mechanisms were suggested by Yang et al. to explain enhanced migration and wound healing of MDA-MB-231 cells [78]. The inflammasome activation by LPS/TLR4 signaling was shown to be mediated by the secretion of HGF [79], a cytokine we found secreted by both cell lines. LPS/TLR4 activation of the ERK pathway was identified as a mechanism of IL-8 [80] and MCP-1 [81,82] activation. Therefore, these findings suggest that low NLRP3-expressed MCF7 loses its migratory features, whereas high NLRP3-expressed MDA-MB-231 cells are more persistent in migration ability upon LPS-ATP-mediated NLRP3 induction.

**Figure 9:**
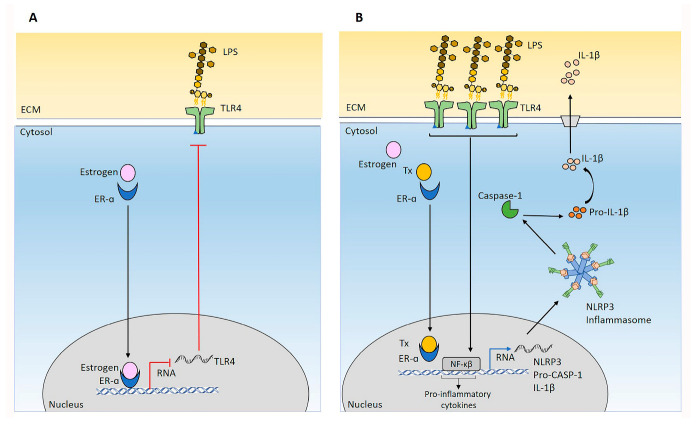
Schematic presentation of Tx mechanisms of NLRP3 activation in MCF7 cells. (**A**) Estrogen binds to the ER-α receptor, which blocks transcription of TLR4 receptor RNA. Blocking TLR4 transcription could result in a reduction of TLR4 expression on the cell membrane. As a result, cells become less susceptible to LPS-induced activation of the inflammasome. (**B**) Tx blocks ER-α. Therefore, the expression of TLR4 becomes enhanced after exposure to LPS, a TLR4 ligand, and an NLRP3 priming signal. This will lead to increased transcription of *NLRP3*, *pro-caspase-1*, and *IL-1β* transcription and protein synthesis. Activation signal (ATP) initiates inflammasome formation and proteolytically cleaves pro-caspase-1. Caspase-1 cleaves pro-IL-1β and creates the release of IL-1β. The secretion of IL-1β from primary BC tumors was shown to repress ER-ɑ in adjacent tumor cells and confers an ER-ɑ- phenotype that causes an aggressive tumor type and Tx resistance [88]. The IL-1β secretion of BC induces chemokine production that could facilitate their interaction with mesenchymal stem cells, enhancing tumor growth or metastasis [89]. In line with this, IL-1β was shown to promote matrix metalloproteinase-9 production and invasion in MCF7 cells [90]. Additionally, our previous study showed that NLRP3-induced IL-1β secretion of MCF7 cells increases the capillary formation of human umbilical vein endothelial cells (HUVECs), which evidenced the angiogenesis and metastasis-promoting effect of IL-1β [9]. Supporting tumor-promoting capacity was the demonstration of IL-1β facilitating BC metastasis to the bone [91,92].

TLR4 was shown to prime NLRP3 activation [83,84] in TNBC cells lines (MDA-MB-231, MDA-MB-468, SUM-149, and SUM-159) compared to ER-α and PR expressing cells (MCF-7, T47D, and CAMA-1) [85]. It was demonstrated that the functional suppression of ER-ɑ using an antagonist Tx could enhance the expression of TLR4 in cancer cells [86]. In addition, blocking estrogen–ER-α interaction by antagonists leads to the accumulation of inflammatory mediators [87]. Estrogen inhibits inflammation induced by LPS, which is mediated through binding to ER [87]. Our data support that LPS/Tx-mediated ER-α suppression induced NLRP3 activation and increased the release of IL-1β in MCF7 cells. These data provide evidence of the possibility of the negative effect of ER-ɑ on NLRP3 activation (Figure 9). Additional analyses of cell lines from the different breast cancer subtypes (luminal A, luminal B, HER2^+^ve, and TNBC) would be worthwhile based on the data generated in this study.

PR inhibition with an antagonist mife was shown to avert LPS-induced NLRP3 activation [93]. Our findings corroborate these data as mife reduced NLRP3 expression in MCF7 cells. Interestingly, co-suppression of ER and PR by Tx/mife reduced NLRP3 expression and IL-1β secretion. It was shown that activation of the ER-α downstream pathway could be modulated by PR in BC [94]. Also, mife blockage of PR could inhibit ER-α signaling [95]. Our data support previous studies showing a regulatory effect of PR inhibition of ER-α pathways. We further advance our understanding of this interaction by demonstrating that blocking PR could reduce NLRP3 expression promoted by ER-α antagonist Tx.

A HER2 antagonist, Tmab, was reported to activate NLRP3 [96]. However, we have found that a functional blockade of HER2 by Tmab had a limited effect on the NLRP3 expression of MCF7 cells. The amplification of HER was not demonstrated in MCF cells [52,97,98], although these cells were shown to have weak to moderate levels of HER2 mRNA and protein expression [52,97,98]. Also, HER2 requires phosphorylation to become active [99]. Tmab mechanisms were shown to be sensitive to HER2 phosphorylation in HER2-negative breast cancers [100,101]. Hence, although the HER2 antagonist effect of Tmab could be limited in MCF7 cells due to a lack of HER2 amplification, our data suggest that suppressing HER2 phosphorylation using Tmab could be insufficient to activate NLRP3.

In conclusion, in this study we demonstrated the high expression of NLRP3 in two TNBC cell lines, MDA-MB-231 and HCC1806, compared to a luminal A group cell line MCF7. Considering the heterogeneous nature of different TNBC tumors, the level of NLRP3 expression could further vary in these cells depending on the difference in their karyotype, microenvironment [102], redox balance [103,104], and osmolarity [105]. Although validations using other ER-α-expressing BC cells appear to be necessary, our findings in MCF7 cells indicate that considerations are required when selecting Tx for treatment of BC patients with ER-α^+^-expressing tumors. This is because Tx treatment in these patients could promote an inflammatory microenvironment, potentially promoting tumor growth. Also, our data further advance our understanding of the mechanisms of NLRP3 activation in hormone-dependent BC tumor cells.

## 4. Material and Methods

### 4.1. The Transcriptomic Profiling of NLRP3 in BC

The Gene Expression Profiling Interactive Analysis (GEPIA) database (http://gepia.cancer-pku.cn/ (accessed on 15 February 2023)) analyzed the NLRP3 transcript level of BC tumors and normal tissues, the effect of NLRP3 in overall and disease-free survival of BC patients, and the correlation between the NLRP3 transcripts and the transcripts of the ER-α coding gene ESR1, the PR encoding gene PGR, and the HER2 encoding gene ERBB2 in the transcriptomic data of BC patients’ tumor samples from the TCGA and GTEx projects. The NLRP3 expression between subgroups of invasive and metastatic BC tumors was compared using the Cancer Genome Atlas Program (TCGA) dataset using the University of Alabama at Birmingham Cancer (UALCAN) portal database (http://ualcan.path.uab.edu/index.html; accessed on 19 February 2023).

The Human Protein Atlas (HPA) version 22.0 (https://www.proteinatlas.org/ (accessed on 15 February 2023)) was used to generate a list of BC cell lines. In addition, the association of the level of NLRP3 and ESR1, PGR, and ERBB2 transcripts was assessed by HPA depending on the molecular portraits of cell line groups, including luminal A, luminal B, Her2^+^, and TNBC.

### 4.2. Cell Lines and Reagents

MCF7, a noninvasive human breast adenocarcinoma cancer cell line (ER-α^+^, PR^+^, and low-level HER2), and MDA-MB-231, HCC1806, an invasive TNBC cell line, were obtained from the American Type Culture Collection (ATCC; Rockville, MD, USA). HCC1806, a TNBC cell line, was kindly gifted by Prof. Sergei Boichuk from Kazan State Medical University. Cells (passage 15) were maintained in Dulbecco’s Modified Eagle’s Medium-F12 (DMEM/F12; PanEco, Moscow, Russia), supplemented with 10% fetal bovine serum (HyClone, Logan, UT, USA), 2 mM L-glutamine (Capricorn Scientific, Darmstadt, Germany), and 1 mM sodium pyruvate (PanEco, Moscow, Russia) in a 5% CO_2_ incubator at 37 °C.

Lipopolysaccharide (LPS) from Escherichia coli O111:B4 (L4391), ATP (A3377), Tx (T5648), and mife (M8046) were purchased from Sigma (St. Louis, MO, USA). Tmab was obtained from Roch Diagnostics GmbH, Mannheim, Germany.

### 4.3. Immunoblotting

Cells were lysed using a Radioimmunoprecipitation Assay buffer to quantify the total protein concentration (Pierce BCA Protein Assay Kit; Thermo Fischer Scientific, Inc., Waltham, MA, USA). Proteins were separated by gel electrophoresis (4–12% two-gradient polyacrylamide gel) and immune-blotted onto PVDF membranes (Biorad, Hercules, CA, USA). Membranes were blocked (5% nonfat milk, 1 h) and incubated with primary antibody rabbit anti-NLRP3 (1:1000, Abcam, Cambridge, MA, USA) overnight at 4 °C. Washed (3×, 5 min with PBS containing 0.1% Tween 20) membranes were incubated with antirabbit IgG (1:2000, Santa Cruz Biotechnology, Germany) secondary antibodies for 2 h at room temperature (RT). A mouse anti-actin Beta-HRP conjugated antibody (1:2000, Sigma, St. Louis, MO, USA) was used to control protein load. Clarity Western ECL Substrate (Bio-Rad, Hercules, CA, USA) was used to reveal the primary–secondary antibody reaction, and signals were detected using ChemiDoc XRS Plus (Bio-Rad, Hercules, CA, USA). The intensity of the signal was quantified using ImageJ software (National Institutes of Health).

### 4.4. Enzyme-Linked Immunosorbent Assay (ELISA)

IL-1β, IL-8, and MCP-1 secretion was analyzed using ELISA following the manufacturer’s recommendations (Vector-Best, Novosibirsk, Russia). Briefly, the cell-free medium (100 µL) was added to anti-IL-1β, anti-IL-8, and anti-MCP-1 antibody precoated wells for 2 h, blocked with 5% bovine serum albumin, and treated with human anti-IL-1β-, anti-IL-8-, and anti-MCP-1-HRP conjugated antibodies for 1 h at RT. Wells were washed (3×; 0.5% Tween20 in PBS) and incubated with 100 µL of substrate solution in the dark for 30 min at RT. The reference standards of IL-1β, IL-8, and MCP-1 were used to generate the standard curve. IL-1β-antibody immune complexes were visualized using a TECAN Infinite 200 plate reader (Grödig, Austria) at OD450 nm and reference OD620 nm. Data were reported as an average of three technical repeats.

### 4.5. Live-Cell Monitoring

MCF-7 and MDA-MB-231 cells were seeded onto an E-Plate 16 (5 × 10^4^ cells per well) (ACEA Biosciences, San Diego, CA, USA) in the complete DMEM/F12 medium. The electrical impedance was recorded every 15 min over 50 h. A cell proliferation curve was generated using the xCELLigence biosensor cell analysis system (ACEA Biosciences, San Diego, CA, USA).

### 4.6. Annexin V Assay

Annexin V expression was assessed using a annexin V: APC assay kit according to the manufacturer’s protocol (BioRad, Hercules, USA). Annexin V positive cells were analyzed by flow cytometry using BD FACSAria III (BD Biosciences, USA). Data were processed using the FlowJo software package (FlowJo LLC, USA). Experiments were conducted in triplicate. Those positive for annexin V-Alexa Flour (AF) 647A only were identified as early apoptotic cells, whereas those positive for both annexin V-AF647A and PI or PI only were categorized as late apoptotic or nonapoptotic cells [106].

### 4.7. Scratch Wound Healing Assay

A confluent monolayer of MDA-MB-231 and MCF7 cells was scratched by dragging a 200 μL pipette tip across the thin membrane and was washed in PBS to remove cell debris, as described by Liang et al. [107]. Monolayers were maintained in the complete DMEM/F12 medium. Microscopic images of the entire wounded area were taken immediately after the scratch, followed by every 24 h for 48 h using an Axiovision Rel 4.5 software with a Zeiss AxioObserver.Z1 microscope. Changes in the wound size were measured using the Axiovision Rel 4.5 software. Each experiment was done in three technical replicates.

### 4.8. Sphere Formation Analysis

To assess the effect of NLRP3 on tumor sphere growth, cells (0.5 × 10^4^) were seeded onto 2.5% Matrigel Basement Membrane Matrix coated 96-well U-bottom plates and maintained in the complete DMEM/F12 medium (5% CO_2_, 37 °C) [108]. Plates were centrifugated at 560× *g* for 10 min to form spheroids. The cells were treated with LPS after 48 h. Sphere size and counts were obtained before LPS (1 µg/mL) treatment (time 0), 30 min, and 24–48 h after the second stimulus. Sphere size and counts were obtained before LPS (1 µg/mL) treatment (time 0), 30 min, and 24–48 h after the second stimulus (ATP 5 mM or Tx 5 µM, mife 10 nM, and Tmab 10 μg/mL) with a Zeiss Observer Z1 inverted microscope (Göttingen, Germany), using Axiovision Rel 4.5 software (Göttingen, Germany).

### 4.9. Cytokine and Chemokine Analysis

Cytokine and chemokine levels were determined using the magnetic bead suspension array using Bio-Plex Pro™ Human Cytokine Screening Panel (48 plex) and Human Chemokine Panel (40 plex) following the manufacturer’s directions (Bio-Rad Laboratories, Hercules, CA, USA). Fifty µL of cell-free culture medium was analyzed by collecting a minimum of 50 beads per analyte. Median fluorescence intensities were collected using a MAGPIX analyzer (Luminex, Austin, TX, USA). Each sample was analyzed in triplicate. Data collected were analyzed with MasterPlex CT control software and MasterPlex QT analysis software (MiraiBio, San Bruno, CA, USA). Standard curves for each cytokine were generated using standards provided by the manufacturer. The average values of measured cytokine and chemokine levels were presented as a heatmap using the web-based tool Heatmapper (http://www.heatmapper.ca/ (accessed on 7 December 2022)) [109].

### 4.10. In Vitro Experimental Design

**Figure 10:**
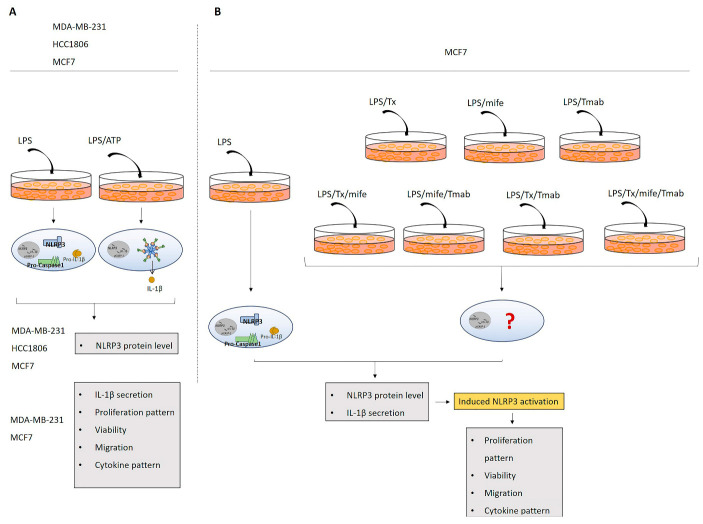
Schematic presentation of the in vitro experimental design. (**A**). LPS-only (3 h) and LPS/ATP (25 min) treatments were used to activate the NLRP3 inflammasome complex in MDA-MB-231, HCC1806, and MCF7 cells. (**B**). The treatment groups: Tx (for 24 h), mife (for 48 h), Tmab (for 48 h), or their combination after pretreatment with LPS for 3 h.

LPS, a PAMP, was used as a priming signal to activate the transcription of NLRP3 and pro-IL-1β [110]. ATP was added to provide an activation signal [111] to form an active inflammasome complex. NLRP3 was activated in MDA-MB-231, HCC1806, and MCF7 cells by adding LPS (1 µg/mL) to the culture medium for 3 h. Washed cells were treated with ATP (5 mM) for 25 min. Receptor antagonists were added 3 h after the end of LPS treatment: ER-ɑ antagonist Tx (5 µM; 24 h), PR antagonist mife (10 nM; 48 h), or HER2 antagonist Tmab (10 μg/mL; 48 h). In some experiments, a combination of Tx/mife, mife/Tmab, Tx/Tmab, or Tx/mife/Tmab was used to block multiple receptors. A schematic of the experimental design is shown in Figure 10.

### 4.11. Statistical Analysis

Statistical analysis was done using IBM SPSS Statistics for Windows, Version 20.0 (IBM Corp., Armonk, NY, USA). One-way ANOVA with Tukey’s post hoc analysis was used to analyze ELISA, sphere formation assays, annexin V, and scratch wound healing assays where the data were parametric. An independent t-test calculated the difference in the scratch wound healing between different cell lines. The Mann–Whitney U test and Kruskal–Wallis one-way analysis of variance were used for the multiplex data where the data were nonparametric. Data are presented as mean  ±  SE. Significance was established at a value of *p*  < 0.05.

## Data Availability

All data generated or analyzed during this study are included in this published article. The data that support the findings of this study are available from the corresponding author upon request.

## Supplementary Materials

The supporting information can be downloaded at: https://www.mdpi.com/article/10.3390/ijms24054846/s1.
