# Supplementary material for: Blocking the Hormone Receptors Modulates NLRP3 in LPS-Primed Breast Cancer Cells
Source: Int J Mol Sci. 2023 Mar 2;24(5):4846. doi: 10.3390/ijms24054846 (PMC10002867; doi:10.3390/ijms24054846)
Supplement: Supplementary file 1 [file ijms-24-04846-s001.zip › ijms-2164366-supplementary.pdf]

Blocking the hormone receptors modulate NLRP3 in LPS-primed breast cancer cells.

Supplementary Figures

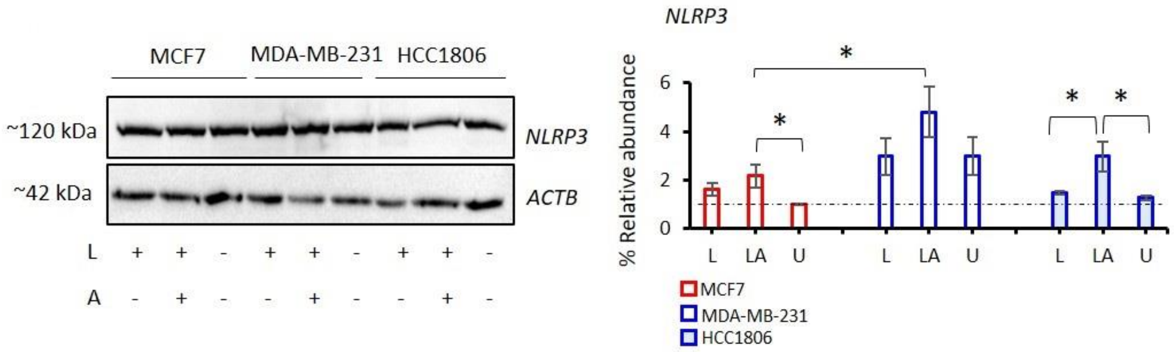

Figure S1. Western blot analysis of NLRP3 protein expression in MDA-MB-231, HCC1806 and MCF7 cells; Data represent two technical repeats. L: LPS-only, LA: LPS/ATP, U: Untreated. P value was calculated using the one-way ANOVA model with Tukey's post hoc tests *p < 0.05.

Supplementary Tables

Table S1. NLRP3 expression in MCF7, MDA-MB-231 and HCC1806 cells after NLRP3 activation

Cell line	Comparison	Mean Difference	Std. E.	P	95% CI Lower	95% CI Upper
MCF7	U-L	-0.62	0.26	0.124	-1.43	0.19
	U-LA	-1.16	0.26	**0.011**	-1.97	-0.35
	L-LA	-0.54	0.26	0.179	-1.35	0.27
MDA-MB-231	U-L	0.02	0.71	1.000	-2.18	2.21
	U-LA	-1.82	0.71	0.096	-4.01	0.37
	L-LA	-1.83	0.71	0.094	-4.03	0.36
HCC1806	U-L	-0.21	0.30	0.780	-1.13	0.72
	U-LA	-1.70	0.30	**0.003**	-2.62	-0.77
	L-LA	-1.49	0.30	**0.006**	-2.42	-0.57

P-value calculated using One Way Anova and Tukey Test. The mean difference is significant at the 0.05 level and showed in bold. **U**: Untreated. **L:** LPS. **LA:** LPS/ATP. n=3 for MCF7 and MDA-MB-231; n=2 for HCC1806.

Table S2. IL-1β secretion of MDA-MB-231 and MCF7 cells after NLRP3 activation

Dependent Variable		Mean Difference	Std. E	P	95% CI Lower Bound	95% CI Upper Bound
MDA-MB-231	U-LA	-78.08	2.20	**<0.001**	-85.14	-71.02
	U-LA	-0.37	2.20	0.998	-7.43	6.69
	LA-L	77.71	2.20	**<0.001**	70.65	84.76
MCF7	U-LA	-0.23	0.11	0.224	-0.59	0.12
	U-LA	0.01	0.11	1.000	-0.34	0.36
	LA-L	0.24	0.11	0.199	-0.11	0.60

P-value calculated using One Way Anova and Tukey Test. The mean difference is significant at the 0.05 level and showed in bold. **U**: Untreated. **L:** LPS. **LA:** LPS/ATP. n=3.

Table S3. The time-dependent changes in tumospheres formed by MDA-MB-231 and MCF7 cells after LPS and LPS/ATP treatments

Cell line	Treatment	Comparison of incubation time	Mean Difference in sphere size	Std. E.	P	95% CI Lower	95% CI Upper
MDA-MB-231	U	0h-4h	112.60	110.89	0.743	-204.66	429.86
		0h-24h	327.40	110.89	**0.042**	10.14	644.66
		0h-48h	552.00	110.89	**0.001**	234.74	869.26
		4h-24h	214.80	110.89	0.252	-102.46	532.06
		4h-48h	439.40	110.89	**0.006**	122.14	756.66
		24h-48h	224.60	110.89	0.220	-92.66	541.86
	L	0h-4h	112.50	260.57	0.972	-661.12	886.12
		0h-24h	221.00	260.57	0.831	-552.62	994.62
		0h-48h	322.00	260.57	0.617	-451.62	1095.62
		4h-24h	108.50	260.57	0.975	-665.12	882.12
		4h-48h	209.50	260.57	0.851	-564.12	983.12
		24h-48h	101.00	260.57	0.979	-672.62	874.62
	LA	0h-4h	-144.00	86.93	0.403	-422.38	134.38
		0h-24h	-400.83	86.93	**0.008**	-679.21	-122.46
		0h-48h	-454.00	86.93	**0.004**	-732.38	-175.62

		4h-24h	-256.83	86.93	**0.071**	-535.21	21.54
		4h-48h	-310.00	86.93	**0.030**	-588.38	-31.62
		24h-48h	-53.17	86.93	0.926	-331.54	225.21
MCF7	U	0h-4h	-15.80	36.03	0.971	-119.65	88.05
		0h-24h	-144.70	38.22	**0.009**	-254.84	-34.56
		0h-48h	-165.01	36.03	**0.002**	-268.86	-61.16
		4h-24h	-128.90	38.22	**0.020**	-239.04	-18.76
		4h-48h	-149.21	36.03	**0.004**	-253.06	-45.36
		24h-48h	-20.31	38.22	0.950	-130.45	89.83
	L	0h-4h	-123.54	37.94	**0.023**	-232.09	-15.00
		0h-24h	-182.74	37.94	**0.001**	-291.29	-74.20
		0h-48h	-188.08	37.94	**0.001**	-296.62	-79.53
		4h-24h	-59.20	37.94	0.427	-167.74	49.34
		4h-48h	-64.53	37.94	0.355	-173.08	44.01
		24h-48h	-5.33	37.94	0.999	-113.88	103.21
	LA	0h-4h	-86.50	31.64	0.062	-176.45	3.45
		0h-24h	-65.75	35.05	0.275	-165.39	33.89
		0h-48h	-138.76	31.64	**0.002**	-228.70	-48.81
		4h-24h	20.75	33.73	0.926	-75.13	116.63
		4h-48h	-52.26	30.17	0.338	-138.02	33.50
		24h-48h	-73.01	33.73	0.173	-168.89	22.88

P-value calculated using One Way Anova and Tukey Test. The mean difference is significant at the 0.05 level and showed in bold. **U:** Untreated. **L:** LPS. **LA:** LPS/ATP. n=5.

Table S4. The comparison of tumosphere size formed by MDA-MB-231 and MCF7 cells upon 48 hours of incubation with LPS or LPS/ATP.

Cell line	Treatment	Mean Difference in sphere size	Std. E.	P	95% CI	
					Lower	Upper
MDA-MB-231	U-L	238.21	127.85	0.205	-118.76	595.18
	U-LA	-392.51	139.19	**0.048**	-781.12	-3.89
	LA-L	-630.72	145.57	**0.005**	-1037.14	-224.29
MCF7	U-L	80.62	20.57	**0.009**	23.18	138.05
	U-LA	-0.23	22.40	1.000	-62.76	62.30
	LA-L	80.84	23.42	**0.018**	15.45	146.24

P-value calculated using One Way Anova and Tukey Test. The mean difference is significant at the 0.05 level and showed in bold. **U:** Untreated. **L**: LPS. **LA:** LPS/ATP. n=5.

Table S5. The effect of LPS-only and LPS/ATP on cell death in BC cells.

Celll ine	Comparisons	Mean Difference	Std. E	P	95% CI	
					Lower Bound	Upper Bound
MDA-MB-231	U-L	-0.32	0.43	0.756	-2.11	1.47
	U-LA	-0.43	0.43	0.623	-2.22	1.36
	L-LA	-0.11	0.43	0.965	-1.90	1.68
MCF7	U-L	-0.86	1.12	0.747	-5.52	3.81
	U-LA	-0.84	1.12	0.756	-5.50	3.83
	L-LA	0.02	1.12	1.000	-4.65	4.69

P-value calculated using One Way Anova and Tukey Test. P values less than 0.05 were considered significant. **U:** Untreated. **L:** LPS. **LA**: LPS/ATP. n=3.

Table S6. The time-dependent changes in recovery of scratched wounded area after LPS and LPS/ATP treatments in BC cells

Cell line	Treatments	Comparisons	Mean Difference	Std. E	P	95% CI	
						Lower	Upper
MDA-MB-231	U	0h-4h	64.25	34.12	0.279	-34.94	163.44

		0h-24h	346.20	32.37	0.000	252.10	440.30
		0h-48h	417.00	32.37	0.000	322.90	511.10
		4h-24h	281.95	32.37	0.000	187.85	376.05
		4h-48h	352.75	32.37	0.000	258.65	446.85
		24h-48h	70.80	30.52	0.141	-17.91	159.51
	L	0h-4h	99.42	32.35	0.039	4.46	194.37
		0h-24h	298.07	30.93	0.000	207.27	388.86
		0h-48h	363.87	30.93	0.000	273.07	454.66
		4h-24h	198.65	28.42	0.000	115.25	282.05
		4h-48h	264.45	28.42	0.000	181.05	347.85
		24h-48h	65.80	26.79	0.115	-12.83	144.43
	LA	0h-4h	31.08	24.55	0.604	-45.56	107.73
		0h-24h	25.00	26.25	0.778	-56.94	106.94
		0h-48h	26.67	26.25	0.745	-55.27	108.61
		4h-24h	-6.08	24.55	0.994	-82.73	70.56
		4h-48h	-4.42	24.55	0.998	-81.06	72.23
		24h-48h	1.67	26.25	1.000	-80.27	83.61
MCF7	U	0h-4h	68.58	44.27	0.441	-62.85	200.01
		0h-24h	194.85	38.88	0.001	79.41	310.29
		0h-48h	200.50	40.99	0.002	78.82	322.18
		4h-24h	126.27	42.33	0.049	0.59	251.94
		4h-48h	131.92	44.27	0.049	0.49	263.35
		24h-48h	5.65	38.88	0.999	-109.79	121.09
	L	0h-4h	49.75	26.60	0.284	-27.57	127.07
		0h-24h	116.85	25.24	0.002	43.49	190.21
		0h-48h	170.85	25.24	0.000	97.49	244.21
		4h-24h	67.10	25.24	0.078	-6.26	140.46
		4h-48h	121.10	25.24	0.001	47.74	194.46
		24h-48h	54.00	23.79	0.153	-15.16	123.16
	LA	0h-4h	6.17	33.01	0.998	-94.82	107.15
		0h-24h	16.50	30.56	0.947	-76.99	109.99
		0h-48h	39.83	33.01	0.637	-61.15	140.82
		4h-24h	10.33	33.01	0.989	-90.65	111.32
		4h-48h	33.67	35.29	0.777	-74.29	141.62
		24h-48h	23.33	33.01	0.892	-77.65	124.32

P-value calculated using One Way Anova and Tukey Test. The mean difference is significant at the 0.05 level and showed in bold. **U:** Untreated**. L:** LPS. **LA:** LPS/ATP. n=5.

Table S7. The comparison of recovery of scratched wounded area of BC cells upon 48 hours of incubation with LPS or LPS/ATP.

Cell line	Incubation time	Comparisons	Mean Difference	Std. E	P	95% CI	
						Lower	Upper
MDA-MB-231	4h	U-L	19.50	43.24	0.895	-101.22	140.22
		U-LA	-43.50	43.24	0.592	-164.22	77.22
		L-LA	-63.00	43.24	0.355	-183.72	57.72
	24h	U-L	-63.80	20.43	**0.027**	-119.80	-7.80
		U-LA	-331.53	23.59	**<0.001**	-396.20	-266.87
		L-LA	-267.73	23.59	**<0.001**	-332.40	-203.07
	48h	U-L	-68.80	11.16	**<0.001**	-99.38	-38.22
		U-LA	-400.67	12.88	**<0.001**	-435.98	-365.35
		L-LA	-331.87	12.88	**<0.001**	-367.18	-296.55
MCF7	4h	U-L	5.17	24.40	0.976	-66.70	77.04
		U-LA	-42.67	26.09	0.294	-119.50	34.17
		L-LA	-47.83	24.40	0.192	-119.70	24.04
	24h	U-L	-54.00	35.01	0.310	-148.55	40.55
		U-LA	-158.60	37.13	**0.003**	-258.88	-58.32
		L-LA	-104.60	37.13	**0.041**	-204.88	-4.32
	48h	U-L	-5.65	31.29	0.982	-93.01	81.71
		U-LA	-140.92	35.63	**0.008**	-240.39	-41.45
		L-LA	-135.27	34.07	**0.008**	-230.38	-40.16

P-value calculated using One Way Anova and Tukey Test. The mean difference is significant at the 0.05 level and showed in bold. **U:** Untreated. **L:** LPS. **LA:** LPS/ATP. n=5.

Table S8. Comparison of cytokine secretion levels in untreated MDA-MB-231 and MCF7 cells.

		Median	SD	SE	Min	Max	Z	P
CTACK	MDA-MB-231	0.33	0.00	0.00	0.33	0.33	0.00	1.000
	MCF7	0.33	0.00	0.00	0.33	0.33		
Eotaxin	MDA-MB-231	0.04	0.02	0.01	0.01	0.04	-1.58	0.114

	MCF7	0.01	0.00	0.00	0.01	0.01		
G-CSF	MDA-MB-231	50.57	429.73	248.10	33.95	786.43	-2.09	**0.037**
	MCF7	0.09	0.00	0.00	0.09	0.09		
GM-CSF	MDA-MB-231	0.66	25.91	14.96	0.07	45.24	-1.55	0.121
	MCF7	0.07	0.00	0.00	0.07	0.07		
GRO-a	MDA-MB-231	21.67	104.26	60.20	21.67	202.26	-1.00	0.317
	MCF7	21.67	0.00	0.00	21.67	21.67		
HGF	MDA-MB-231	3.79	2.13	1.23	0.10	3.79	-0.90	0.369
	MCF7	0.45	1.02	0.59	0.10	2.02		
IFN-a2	MDA-MB-231	0.48	0.00	0.00	0.48	0.48	0.00	1.000
	MCF7	0.48	0.00	0.00	0.48	0.48		
IFN-g	MDA-MB-231	0.56	0.29	0.17	0.18	0.74	-1.53	0.127
	MCF7	0.14	0.23	0.13	0.02	0.46		
IL-10	MDA-MB-231	0.14	0.00	0.00	0.14	0.14	0.00	1.000
	MCF7	0.14	0.00	0.00	0.14	0.14		
IL-12p40	MDA-MB-231	1.92	29.50	17.03	1.92	53.01	-1.00	0.317
	MCF7	1.92	0.00	0.00	1.92	1.92		
IL-12p70	MDA-MB-231	0.12	0.00	0.00	0.12	0.12	0.00	1.000
	MCF7	0.12	0.00	0.00	0.12	0.12		
IL-13	MDA-MB-231	0.05	0.05	0.03	0.05	0.14	-0.26	0.796
	MCF7	0.05	0.18	0.11	0.05	0.37		
IL-15	MDA-MB-231	5.80	0.00	0.00	5.80	5.80	0.00	1.000
	MCF7	5.80	0.00	0.00	5.80	5.80		
IL-16	MDA-MB-231	0.23	3.26	1.88	0.13	5.83	-1.55	0.121
	MCF7	0.13	0.00	0.00	0.13	0.13		
IL-17	MDA-MB-231	0.23	0.00	0.00	0.23	0.23	0.00	1.000
	MCF7	0.23	0.00	0.00	0.23	0.23		
IL-18	MDA-MB-231	1.04	1.46	0.84	0.63	3.34	-2.09	**0.037**
	MCF7	0.16	0.00	0.00	0.16	0.16		
IL-1Ra	MDA-MB-231	52.62	143.25	82.71	9.23	276.18	-1.55	0.121
	MCF7	9.23	0.00	0.00	9.23	9.23		
IL-1a	MDA-MB-231	1.73	1.41	0.82	0.04	2.85	-1.16	0.246
	MCF7	0.04	0.16	0.09	0.04	0.31		
IL-1b	MDA-MB-231	0.01	0.06	0.04	0.01	0.12	-0.26	0.796
	MCF7	0.01	0.01	0.00	0.01	0.02		
IL-2	MDA-MB-231	0.52	0.00	0.00	0.52	0.52	0.00	1.000
	MCF7	0.52	0.00	0.00	0.52	0.52		
IL-2Ra	MDA-MB-231	0.14	0.00	0.00	0.14	0.14	-1.00	0.317
	MCF7	0.14	0.59	0.34	0.14	1.17		
IL-3	MDA-MB-231	0.02	0.02	0.01	0.01	0.04	-0.94	0.346
	MCF7	0.01	0.01	0.00	0.01	0.02		
IL-4	MDA-MB-231	0.14	0.00	0.00	0.14	0.14	0.00	1.000
	MCF7	0.14	0.00	0.00	0.14	0.14		
IL-5	MDA-MB-231	1.13	0.00	0.00	1.13	1.13	0.00	1.000
	MCF7	1.13	0.00	0.00	1.13	1.13		
IL-6	MDA-MB-231	11.33	45.46	26.25	0.30	83.98	-1.55	0.121
	MCF7	0.30	0.00	0.00	0.30	0.30		
IL-7	MDA-MB-231	0.35	1.23	0.71	0.17	2.39	-1.55	0.121
	MCF7	0.17	0.00	0.00	0.17	0.17		
IL-8	MDA-MB-231	0.26	1.84	1.06	0.26	3.45	-1.00	0.317
	MCF7	0.26	0.00	0.00	0.26	0.26		
IL-9	MDA-MB-231	0.05	0.00	0.00	0.05	0.05	0.00	1.000
	MCF7	0.05	0.00	0.00	0.05	0.05		
IP10	MDA-MB-231	2.40	39.68	22.91	2.11	70.98	-1.96	**0.050**
	MCF7	0.70	0.81	0.47	0.44	1.96		
LIF	MDA-MB-231	3.29	12.63	7.29	3.29	25.16	-1.00	0.317
	MCF7	3.29	0.00	0.00	3.29	3.29		
M-CSF	MDA-MB-231	1.28	1.21	0.70	0.01	2.42	-1.55	0.121
	MCF7	0.01	0.00	0.00	0.01	0.01		
MCP-1	MDA-MB-231	1.58	0.69	0.40	0.56	1.88	-2.09	**0.037**
	MCF7	0.11	0.00	0.00	0.11	0.11		
MCP-3	MDA-MB-231	0.06	0.13	0.07	0.02	0.26	-1.55	0.121
	MCF7	0.02	0.00	0.00	0.02	0.02		
MIF	MDA-MB-231	23.53	73.52	42.45	1.68	138.53	-0.22	0.827
	MCF7	28.94	35.92	20.74	8.12	78.07		
MIG	MDA-MB-231	0.04	0.00	0.00	0.04	0.04	0.00	1.000
	MCF7	0.04	0.00	0.00	0.04	0.04		
MIP-1a	MDA-MB-231	0.26	0.10	0.06	0.19	0.39	-2.09	**0.037**
	MCF7	0.03	0.00	0.00	0.03	0.03		
MIP-1b	MDA-MB-231	1.05	1.27	0.73	0.20	2.70	0.00	1.000
	MCF7	0.87	3.02	1.75	0.20	5.74		
PDGF-BB	MDA-MB-231	0.24	0.00	0.00	0.24	0.24	-1.00	0.317

	MCF7	0.24	1.28	0.74	0.24	2.45		
RANTES	MDA-MB-231	0.01	1.53	0.88	0.01	2.66	-0.26	0.796
	MCF7	0.01	0.84	0.48	0.01	1.46		
SCF	MDA-MB-231	0.50	0.99	0.57	0.50	2.21	-2.12	**0.034**
	MCF7	0.02	0.00	0.00	0.02	0.02		
SCGF-b	MDA-MB-231	7.82	0.00	0.00	7.82	7.82	0.00	1.000
	MCF7	7.82	0.00	0.00	7.82	7.82		
SDF-1a	MDA-MB-231	1.52	0.91	0.52	1.52	3.09	-0.26	0.796
	MCF7	1.52	1.20	0.69	1.52	3.59		
TNF-a	MDA-MB-231	0.14	36.44	21.04	0.14	63.25	-1.00	0.317
	MCF7	0.14	0.00	0.00	0.14	0.14		
TNF-b	MDA-MB-231	0.30	0.00	0.00	0.30	0.30	0.00	1.000
	MCF7	0.30	0.00	0.00	0.30	0.30		
TRAIL	MDA-MB-231	0.10	0.02	0.01	0.07	0.10	-1.29	0.197
	MCF7	0.10	0.40	0.23	0.10	0.80		
VEGF	MDA-MB-231	5.35	2.59	1.50	5.35	9.84	-1.00	0.317
	MCF7	5.35	0.00	0.00	5.35	5.35		
b-NGF	MDA-MB-231	0.85	0.00	0.00	0.85	0.85	0.00	1.000
	MCF7	0.85	0.00	0.00	0.85	0.85		
bFGF	MDA-MB-231	5.55	3.43	1.98	1.02	7.75	-1.55	0.121
	MCF7	1.02	0.00	0.00	1.02	1.02		

SD: Standard deviation, **SE:** Standard error, **Min:** Minimum, **Max:** maximum.

P value was calculated using Mann-Whitney U test. P values less than 0.05 was shown in bold. **U:** Untreated, **LA:** LPS/ATP (n=3)

Table S9. IL-8 and MCP-1 secretion of MDA-MB-231 and MCF7 cells after NLRP3 activation

		t-test for Equality of Means						
							95% CI	
		t	df	P	Mean Difference	Std. E.	Lower	Upper
IL-8	MCF-7	-6.72	4	**0.003**	-2.36	0.35	-3.33	-1.38
	MDA-MB-231	-21.06	4	**<0.001**	-4.39	0.21	-4.96	-3.81
MCP-1	MCF-7	-5.44	4	**0.006**	-2.37	0.44	-3.58	-1.16
	MDA-MB-231	-21.43	4	**<0.001**	-8.44	0.39	-9.54	-7.35

P-value calculated using an Independent Sample T Test. The mean difference is significant at the 0.05 level and showed in bold. U: Untreated. L: LPS. LA: LPS/ATP. n=3.

Table S10. Cytokine secretion levels in MDA-MB-231 cells after LPS-only and LPS/ATP treatments.

		Median	SD	SE	Min	Max	Z	P
CTACK	U	0.33	0.00	0.00	0.33	0.33	-2.09	**0.037**
	LA	4.54	0.85	0.49	4.09	5.73		
Eotaxin	U	0.04	0.02	0.01	0.01	0.04	-0.90	0.369
	LA	0.14	0.10	0.06	0.01	0.20		
G-CSF	U	50.57	429.73	248.10	33.95	786.43	-1.53	0.127
	LA	2318.10	20175.85	11648.53	143.96	36125.87		
GM-CSF	U	0.66	25.91	14.96	0.07	45.24	-0.44	0.658
	LA	11.83	165.92	95.79	0.07	293.15		
GRO-a	U	21.67	104.26	60.20	21.67	202.26	-1.16	0.246
	LA	2819.67	1663.83	960.61	21.67	2980.59		
HGF	U	3.79	2.13	1.23	0.10	3.79	-1.99	**0.046**
	LA	8.82	3.88	2.24	4.59	12.34		
IFN-a2	U	0.48	0.00	0.00	0.48	0.48	-1.00	0.317
	LA	0.48	0.13	0.07	0.48	0.70		
IFN-g	U	0.56	0.29	0.17	0.18	0.74	-1.09	0.275
	LA	1.00	0.69	0.40	0.45	1.83		
IL-10	U	0.14	0.00	0.00	0.14	0.14	-1.55	0.121
	LA	0.60	5.02	2.90	0.14	9.05		
IL-12p40	U	1.92	29.50	17.03	1.92	53.01	-0.23	0.817
	LA	4.23	15.40	8.89	1.92	29.67		
IL-12p70	U	0.12	0.00	0.00	0.12	0.12	-1.00	0.317
	LA	0.12	0.77	0.45	0.12	1.46		
IL-13	U	0.05	0.05	0.03	0.05	0.14	-0.70	0.487
	LA	0.08	0.67	0.39	0.05	1.22		
IL-15	U	5.80	0.00	0.00	5.80	5.80	-1.55	0.121
	LA	51.86	36.71	21.19	5.80	78.34		
IL-16	U	0.23	3.26	1.88	0.13	5.83	-0.65	0.513
	LA	1.99	1.58	0.91	1.52	4.46		
IL-17	U	0.23	0.00	0.00	0.23	0.23	-1.55	0.121
	LA	0.31	0.32	0.18	0.23	0.82		

IL-18	U	1.04	1.46	0.84	0.63	3.34	-1.09	0.275
	LA	3.20	544.83	314.56	2.26	946.40		
IL-1Ra	U	52.62	143.25	82.71	9.23	276.18	-0.44	0.658
	LA	19.59	27.72	16.00	9.23	61.57		
IL-1a	U	1.73	1.41	0.82	0.04	2.85	-1.96	**0.050**
	LA	3.22	0.33	0.19	3.14	3.74		
IL-1b	U	0.01	0.06	0.04	0.01	0.12	-1.99	**0.046**
	LA	0.43	1.47	0.85	0.33	2.93		
IL-2	U	0.52	0.00	0.00	0.52	0.52	-1.55	0.121
	LA	1.91	1.69	0.97	0.52	3.88		
IL-2Ra	U	0.14	0.00	0.00	0.14	0.14	-1.00	0.317
	LA	0.14	1.22	0.70	0.14	2.25		
IL-3	U	0.02	0.02	0.01	0.01	0.04	-1.96	**0.050**
	LA	0.11	80.64	46.56	0.05	139.75		
IL-4	U	0.14	0.00	0.00	0.14	0.14	-1.55	0.121
	LA	0.58	0.29	0.17	0.14	0.68		
IL-5	U	1.13	0.00	0.00	1.13	1.13	-1.55	0.121
	LA	21.04	17.53	10.12	1.13	36.07		
IL-6	U	11.33	45.46	26.25	0.30	83.98	-0.65	0.513
	LA	75.79	1516.63	875.63	6.77	2667.48		
IL-7	U	0.35	1.23	0.71	0.17	2.39	0.00	1.000
	LA	1.70	0.92	0.53	0.17	1.82		
IL-8	U	0.26	1.84	1.06	0.26	3.45	-1.99	**0.046**
	LA	18.41	3545.72	2047.12	4.42	6152.77		
IL-9	U	0.05	0.00	0.00	0.05	0.05	-2.09	**0.037**
	LA	6.66	3.23	1.87	2.68	9.08		
IP10	U	2.40	39.68	22.91	2.11	70.98	-1.09	0.275
	LA	22.63	259.73	149.96	14.41	468.33		
LIF	U	3.29	12.63	7.29	3.29	25.16	-1.55	0.121
	LA	60.52	36.24	20.92	21.54	93.94		
M-CSF	U	1.28	1.21	0.70	0.01	2.42	-1.96	**0.050**
	LA	23.85	11.33	6.54	8.76	30.94		
MCP-1	U	1.58	0.69	0.40	0.56	1.88	-1.96	**0.050**
	LA	8.75	3.74	2.16	7.77	14.69		
MCP-3	U	0.06	0.13	0.07	0.02	0.26	-0.44	0.658
	LA	0.17	2.12	1.23	0.02	3.77		
MIF	U	23.53	73.52	42.45	1.68	138.53	-0.22	0.827
	LA	20.00	222.00	128.17	2.90	395.68		
MIG	U	0.04	0.00	0.00	0.04	0.04	-1.55	0.121
	LA	1.42	1.66	0.96	0.04	3.34		
MIP-1a	U	0.26	0.10	0.06	0.19	0.39	-1.96	**0.050**
	LA	0.47	0.06	0.03	0.41	0.53		
MIP-1b	U	1.05	1.27	0.73	0.20	2.70	-0.44	0.658
	LA	1.64	1.52	0.88	0.20	3.23		
PDGF-BB	U	0.24	0.00	0.00	0.24	0.24	0.00	1.000
	LA	0.24	0.00	0.00	0.24	0.24		
RANTES	U	0.01	1.53	0.88	0.01	2.66	-1.99	**0.046**
	LA	11.24	5.25	3.03	4.65	15.02		
SCF	U	0.50	0.99	0.57	0.50	2.21	-1.99	**0.046**
	LA	7.52	2.83	1.63	3.85	9.42		
SCGF-b	U	7.82	0.00	0.00	7.82	7.82	-1.00	0.317
	LA	7.82	79.83	46.09	7.82	146.09		
SDF-1a	U	1.52	0.91	0.52	1.52	3.09	-0.26	0.796
	LA	1.52	0.98	0.56	1.52	3.21		
TNF-a	U	0.14	36.44	21.04	0.14	63.25	-0.66	0.507
	LA	3.36	7.79	4.50	2.48	16.39		
TNF-b	U	0.30	0.00	0.00	0.30	0.30	-1.55	0.121
	LA	1.63	5.49	3.17	0.30	10.41		
TRAIL	U	0.10	0.02	0.01	0.07	0.10	-1.62	0.105
	LA	0.24	0.47	0.27	0.10	0.97		
VEGF	U	5.35	2.59	1.50	5.35	9.84	-0.26	0.796
	LA	5.35	133.67	77.18	5.35	236.88		
b-NGF	U	0.85	0.00	0.00	0.85	0.85	0.00	1.000
	LA	0.85	0.00	0.00	0.85	0.85		
bFGF	U	5.55	3.43	1.98	1.02	7.75	-1.33	0.184
	LA	10.78	7.79	4.50	5.55	20.87		

SD: Standard deviation, **SE:** Standard error, **Min:** Minimum, **Max:** maximum.

P value was calculated using Mann-Whitney U test. P values less than 0.05 was shown in bold. **U:** Untreated, **LA:** LPS/ATP (n=3)

Table S11: Cytokine secretion levels in MCF7 cells after LPS-only and LPS/ATP treatments.

		Median	SD	SE	Min	Max	Z	P
CTACK	U	0.33	0.00	0.00	0.33	0.33	0.00	1.000
	LA	0.33	0.00	0.00	0.33	0.33		
Eotaxin	U	0.01	0.00	0.00	0.01	0.01	-1.00	0.317
	LA	0.01	0.20	0.11	0.01	0.35		
G-CSF	U	0.09	0.00	0.00	0.09	0.09	-1.00	0.317
	LA	0.09	4.52	2.61	0.09	7.92		
GM-CSF	U	0.07	0.00	0.00	0.07	0.07	-1.00	0.317
	LA	0.07	0.18	0.10	0.07	0.38		
GRO-a	U	21.67	0.00	0.00	21.67	21.67	0.00	1.000
	LA	21.67	0.00	0.00	21.67	21.67		
HGF	U	0.45	1.02	0.59	0.10	2.02	-0.89	0.376
	LA	1.88	0.20	0.12	1.62	2.02		
IFN-a2	U	0.48	0.00	0.00	0.48	0.48	0.00	1.000
	LA	0.48	0.00	0.00	0.48	0.48		
IFN-g	U	0.14	0.23	0.13	0.02	0.46	-0.44	0.658
	LA	0.13	0.17	0.10	0.02	0.36		
IL-10	U	0.14	0.00	0.00	0.14	0.14	0.00	1.000
	LA	0.14	0.00	0.00	0.14	0.14		
IL-12p40	U	1.92	0.00	0.00	1.92	1.92	-1.00	0.317
	LA	1.92	13.49	7.79	1.92	25.29		
IL-12p70	U	0.12	0.00	0.00	0.12	0.12	0.00	1.000
	LA	0.12	0.00	0.00	0.12	0.12		
IL-13	U	0.05	0.18	0.11	0.05	0.37	-1.00	0.317
	LA	0.05	0.00	0.00	0.05	0.05		
IL-15	U	5.80	0.00	0.00	5.80	5.80	0.00	1.000
	LA	5.80	0.00	0.00	5.80	5.80		
IL-16	U	0.13	0.00	0.00	0.13	0.13	0.00	1.000
	LA	0.13	0.00	0.00	0.13	0.13		
IL-17	U	0.23	0.00	0.00	0.23	0.23	0.00	1.000
	LA	0.23	0.00	0.00	0.23	0.23		
IL-18	U	0.16	0.00	0.00	0.16	0.16	0.00	1.000
	LA	0.16	0.00	0.00	0.16	0.16		
IL-1Ra	U	9.23	0.00	0.00	9.23	9.23	-1.58	0.114
	LA	14.86	3.25	1.88	9.23	14.86		
IL-1a	U	0.04	0.16	0.09	0.04	0.31	-1.00	0.317
	LA	0.04	0.00	0.00	0.04	0.04		
IL-1b	U	0.01	0.01	0.00	0.01	0.02	-1.00	0.317
	LA	0.01	0.00	0.00	0.01	0.01		
IL-2	U	0.52	0.00	0.00	0.52	0.52	0.00	1.000
	LA	0.52	0.00	0.00	0.52	0.52		
IL-2Ra	U	0.14	0.59	0.34	0.14	1.17	-1.00	0.317
	LA	0.14	0.00	0.00	0.14	0.14		
IL-3	U	0.01	0.01	0.00	0.01	0.02	-1.16	0.246
	LA	0.03	0.03	0.02	0.01	0.07		
IL-4	U	0.14	0.00	0.00	0.14	0.14	0.00	1.000
	LA	0.14	0.00	0.00	0.14	0.14		
IL-5	U	1.13	0.00	0.00	1.13	1.13	0.00	1.000
	LA	1.13	0.00	0.00	1.13	1.13		
IL-6	U	0.30	0.00	0.00	0.30	0.30	0.00	1.000
	LA	0.30	0.00	0.00	0.30	0.30		
IL-7	U	0.17	0.00	0.00	0.17	0.17	0.00	1.000
	LA	0.17	0.00	0.00	0.17	0.17		
IL-8	U	0.26	0.00	0.00	0.26	0.26	-1.00	0.317
	LA	0.26	2.06	1.19	0.26	3.83		
IL-9	U	0.05	0.00	0.00	0.05	0.05	0.00	1.000
	LA	0.05	0.00	0.00	0.05	0.05		
IP10	U	0.70	0.81	0.47	0.44	1.96	0.00	1.000
	LA	0.92	0.56	0.32	0.44	1.56		
LIF	U	3.29	0.00	0.00	3.29	3.29	-2.09	**0.037**
	LA	26.83	0.84	0.48	26.01	27.68		
M-CSF	U	0.01	0.00	0.00	0.01	0.01	-1.00	0.317
	LA	0.01	0.43	0.25	0.01	0.75		
MCP-1	U	0.11	0.00	0.00	0.11	0.11	-1.00	0.317
	LA	0.11	0.19	0.11	0.11	0.44		
MCP-3	U	0.02	0.00	0.00	0.02	0.02	-1.55	0.121
	LA	0.13	0.24	0.14	0.02	0.48		
MIF	U	28.94	35.92	20.74	8.12	78.07	-0.65	0.513
	LA	3.62	156.11	90.13	1.68	273.04		
MIG	U	0.04	0.00	0.00	0.04	0.04	0.00	1.000

	LA	0.04	0.00	0.00	0.04	0.04		
MIP-1a	U	0.03	0.00	0.00	0.03	0.03	-1.00	0.317
	LA	0.03	0.02	0.01	0.03	0.06		
MIP-1b	U	0.87	3.02	1.75	0.20	5.74	-1.55	0.121
	LA	0.20	0.00	0.00	0.20	0.20		
PDGF-BB	U	0.24	1.28	0.74	0.24	2.45	-0.26	0.796
	LA	0.24	8.55	4.94	0.24	15.05		
RANTES	U	0.01	0.84	0.48	0.01	1.46	-1.00	0.317
	LA	0.01	0.00	0.00	0.01	0.01		
SCF	U	0.02	0.00	0.00	0.02	0.02	0.00	1.000
	LA	0.02	0.00	0.00	0.02	0.02		
SCGF-b	U	7.82	0.00	0.00	7.82	7.82	-1.00	0.317
	LA	7.82	92.16	53.21	7.82	167.45		
SDF-1a	U	1.52	1.20	0.69	1.52	3.59	-0.70	0.487
	LA	2.96	1.51	0.87	1.52	4.54		
TNF-a	U	0.14	0.00	0.00	0.14	0.14	0.00	1.000
	LA	0.14	0.00	0.00	0.14	0.14		
TNF-b	U	0.30	0.00	0.00	0.30	0.30	0.00	1.000
	LA	0.30	0.00	0.00	0.30	0.30		
TRAIL	U	0.10	0.40	0.23	0.10	0.80	-1.00	0.317
	LA	0.10	0.00	0.00	0.10	0.10		
VEGF	U	5.35	0.00	0.00	5.35	5.35	0.00	1.000
	LA	5.35	0.00	0.00	5.35	5.35		
b-NGF	U	0.85	0.00	0.00	0.85	0.85	0.00	1.000
	LA	0.85	0.00	0.00	0.85	0.85		
bFGF	U	1.02	0.00	0.00	1.02	1.02	-1.00	0.317
	LA	1.02	0.88	0.51	1.02	2.54		

SD: Standard deviation, **SE:** Standard error, **Min:** Minimum, **Max:** maximum.

P value was calculated using Mann-Whitney U test. P values less than 0.05 was shown in bold. **U:** Untreated, **LA:** LPS/ATP (n=3)

Table S12. NLRP3 expression in MCF7 cells after tamoxifen, mifepristone, and trastuzumab and their combined treatments

Comparisons	Mean Difference	Std. E.	P	95% CI	
				Lower	Upper
L/L-Tx	-0,27	0,07	0,050	-0,54	0,00
L/L-mife	0,19	0,07	0,250	-0,09	0,46
L/L-Tmab	-0,05	0,07	0,988	-0,33	0,22
L/L-Tx-Mife	0,05	0,07	0,996	-0,23	0,32
L/L-Mife-Tmab	0,02	0,07	1,000	-0,25	0,29
L/L-Tx-Tmab	-0,28	0,07	0,044	-0,55	-0,01
L/L-Tx-Mife-Tmab	0,24	0,07	0,101	-0,04	0,51
L-Tx/L-mife	0,46	0,07	0,002	0,18	0,73
L-Tx/L-Tmab	0,22	0,07	0,146	-0,06	0,49
L-Tx/L-Tx-Mife	0,32	0,07	0,023	0,04	0,59
L-Tx/L-Mife-Tmab	0,29	0,07	0,036	0,02	0,56
L-Tx/L-Tx-Tmab	-0,01	0,07	1,000	-0,28	0,26
L-Tx/L-Tx-Mife-Tmab	0,51	0,07	0,001	0,23	0,78
L-mife/L-Tmab	-0,24	0,07	0,092	-0,51	0,03
L-mife/L-Tx-Mife	-0,14	0,07	0,516	-0,41	0,13
L-mife/L-Mife-Tmab	-0,17	0,07	0,352	-0,44	0,11
L-mife/L-Tx-Tmab	-0,47	0,07	0,002	-0,74	-0,19
L-mife/L-Tx-Mife-Tmab	0,05	0,07	0,993	-0,22	0,32

L-Tmab/L-Tx-Mife	0,10	0,07	0,813	-0,17	0,37
L-Tmab/L-Mife-Tmab	0,08	0,07	0,943	-0,20	0,35
L-Tmab/L-Tx-Tmab	-0,23	0,07	0,121	-0,50	0,05
L-Tmab/L-Tx-Mife-Tmab	0,29	0,07	0,036	0,02	0,56
L-Tx-Mife/L-Mife-Tmab	-0,03	0,07	1,000	-0,30	0,25
L-Tx-Mife/L-Tx-Tmab	-0,33	0,07	0,020	-0,60	-0,05
L-Tx-Mife/L-Tx-Mife-Tmab	0,19	0,07	0,229	-0,08	0,46
L-Mife-Tmab/L-Tx-Tmab	-0,30	0,07	0,030	-0,57	-0,03
L-Mife-Tmab/L-Tx-Mife-Tmab	0,22	0,07	0,146	-0,06	0,49
L-Tx-Tmab/L-Tx-Mife-Tmab	0,52	0,07	0,001	0,24	0,79

P-value calculated using One Way Anova and Tukey Test. The mean difference is significant at the 0.05 level and showed in bold. U: Untreated. L: LPS. LA: LPS/ATP. n=3.

Table S13. IL-1β secretion of LPS primed MCF7 cells after tamoxifen, mifepristone, and trastuzumab and their combined treatments.

	Mean Difference	Std. E	P	95% CI	
				Lower	Upper
L-L/Tx	-0.89	0.23	**0.027**	-1.70	-0.08
L-L/mife	0.96	0.23	**0.015**	0.14	1.77
L-L/Tmab	0.70	0.23	0.115	-0.11	1.52
L-L/Tx/Mife	0.75	0.23	0.083	-0.06	1.56
L-L/mife/Tmab	0.03	0.23	1.000	-0.78	0.84
L-L/Tx/Tmab	-0.20	0.23	0.985	-1.02	0.61
L-L/Tx/mife/Tmab	0.03	0.23	1.000	-0.78	0.84
L-Lx-L/mife	1.85	0.23	**<0.001**	1.03	2.66
L/Tx-L/Tmab	1.59	0.23	**<0.001**	0.78	2.40
L/Tx-L/Tx/Mife	1.64	0.23	**<0.001**	0.83	2.45
L/Tx-L/mife/Tmab	0.92	0.23	**0.021**	0.11	1.73
L/Tx-L/Tx/Tmab	0.69	0.23	0.132	-0.13	1.50
L/Tx-L/Tx/mife/Tmab	0.92	0.23	**0.021**	0.11	1.73
L/mife-L/Tmab	-0.25	0.23	0.953	-1.06	0.56
L/mife-L/Tx/Mife	-0.21	0.23	0.983	-1.02	0.60
L/mife-L/mife/Tmab	-0.93	0.23	**0.020**	-1.74	-0.12
L/mife-L/Tx/Tmab	-1.16	0.23	**0.003**	-1.97	-0.35
L/mife-L/Tx/mife/Tmab	-0.93	0.23	**0.020**	-1.74	-0.12
L/Tmab--L/Tx/Mife	0.04	0.23	1.000	-0.77	0.86
L/Tmab-L/mife/Tmab	-0.67	0.23	0.143	-1.49	0.14

L/Tmab-L/Tx/Tmab	-0.91	0.23	**0.023**	-1.72	-0.10
L/Tmab-L/Tx/mife/Tmab	-0.67	0.23	0.143	-1.49	0.14
L/Tx/Mife-L/mife/Tmab	-0.72	0.23	0.103	-1.53	0.09
L/Tx/Mife-L/Tx/Tmab	-0.95	0.23	**0.016**	-1.76	-0.14
L/Tx/Mife-L/Tx/mife/Tmab	-0.72	0.23	0.103	-1.53	0.09
L/mife/Tmab-L/Tx/Tmab	-0.23	0.23	0.969	-1.04	0.58
L/mife/Tmab-L/Tx/mife/Tmab	0.00	0.23	1.000	-0.81	0.81
L/Tx/Tmab-L/Tx/mife/Tmab	0.23	0.23	0.969	-0.58	1.04

P-value calculated using One Way Anova and Tukey Test. The mean difference is significant at the 0.05 level and showed in bold. U: Untreated. L: LPS. LA: LPS/ATP. n=3.

Table S14. The time-dependent changes in tumospheres formed by MCF7 cells after LPS/Tamoxifen treatment

Cell line	Treatment	Comparison of incubation time	Mean Difference in sphere size	Std. E.	P	95% CI	
						Lower	Upper
MCF7	LTx	0h-4h	526.32	38.62	**<0.001**	418.21	634.42
		0h-24h	-107.84	38.62	0.051	-215.94	0.27
		0h-48h	-147.63	38.62	**0.005**	-255.73	-39.52
		4h-24h	-634.15	38.62	**<0.001**	-742.25	-526.05
		4h-48h	-673.94	38.62	**<0.001**	-782.04	-565.84
		24h-48h	-39.79	38.62	0.734	-147.89	68.31

P-value calculated using One Way Anova and Tukey Test. The mean difference is significant at the 0.05 level and showed in bold. **LTx:** LPS/Tamoxifen. n=5.

Table S15. The comparison of tumosphere size formed by MCF7 cells upon 4, 24 and 48 hours of incubation with LPS, LPS/ATP or LPS/Tamoxifen.

Cell line	Incubation time	Treatment	Mean Difference in sphere size	Std. E.	P	95% CI	
						Lower	Upper
MCF7	4h	L-LA	19.90	31.37	0.804	-62.21	102.01
		L-LTx	489.47	31.37	**<0.001**	407.36	571.58
		LA-LTx	469.57	29.91	**<0.001**	391.28	547.86
	24h	L-LA	99.85	49.33	0.149	-31.75	231.45
		L-LTx	-85.48	44.53	0.175	-204.27	33.31
		LA-LTx	-185.33	47.46	**0.005**	-311.96	-58.70
	48h	L-LA	32.18	36.39	0.659	-63.07	127.42
		L-LTx	-119.94	36.39	**0.014**	-215.18	-24.69
		LA-LTx	-152.12	34.70	**0.002**	-242.93	-61.30

P-value calculated using One Way Anova and Tukey Test. The mean difference is significant at the 0.05 level and showed in bold. **LTx:** LPS/Tamoxifen. n=5.

Table S16. The time-dependent changes in recovery of scratched wounded area of MCF7 cells after LPS/Tamoxifen treatment

	Treatment	Comparison of the incubation time	Mean Difference	Std. E	P	95% CI	
						Lower	Upper
MCF7	LTx	0h-4h	22.21	18.26	0.628	-31.39	75.81
		0h-24h	30.82	19.88	0.438	-27.54	89.17
		0h-48h	179.77	17.22	**<0.001**	129.24	230.31
		4h-24h	8.61	20.79	0.975	-52.42	69.63
		4h-48h	157.56	18.26	**<0.001**	103.96	211.16
		24h-48h	148.96	19.88	**<0.001**	90.61	207.31

P-value calculated using One Way Anova and Tukey Test. The mean difference is significant at the 0.05 level and showed in bold. **LTx:** LPS/Tamoxifen. n=5.

Table S17. The recovery of scratched wounded area in MCF7 cells upon 4, 24 and 48 hours of incubation with LPS, LPS/ATP or LPS/Tamoxifen.

Cell line	Incubation time	Comparisons	Mean Difference	Std. E	P	95% CI	
						Lower	Upper
MCF7	4h	L-LA	-47.83	24.38	0.177	-115.90	20.23
		L-LTx	8.17	21.41	0.923	-51.61	67.95
		LA-LTx	56.01	23.31	0.091	-9.07	121.09
	24h	L-LA	-104.60	27.72	**0.011**	-182.00	-27.20
		L-LTx	-53.97	30.18	0.228	-138.23	30.29
		LA-LTx	50.63	31.56	0.293	-37.49	138.75
	48h	L-LA	-135.27	27.63	**0.002**	-211.02	-59.52
		L-LTx	40.99	23.93	0.248	-24.61	106.59
		LA-LTx	176.25	27.63	**<0.001**	100.50	252.01

P-value calculated using One Way Anova and Tukey Test. The mean difference is significant at the 0.05 level and showed in bold. **.L:** LPS; **LA:** LPS/ATP; **LTx:** LPS/Tamoxifen. n=5.

Table S18. Cytokine secretion levels in MCF7 cells after LPS-only, LPS/ATP and LPS/Tamoxifen treatments.

		Median	Std. Deviation	Std. Error of Mean	Minimum	Maximum	P		Z	P
HGF	U	0.45	1.02	0.59	0.10	2.02	0.035	U-L	-0.89	0.376
	L	1.88	0.20	0.12	1.62	2.02		U-LA	-1.96	**0.050**
	LA	3.34	0.02	0.01	3.32	3.36		U-LTx	-1.62	0.105
	LTx	2.02	0.20	0.11	2.02	2.36		L-LA	-1.96	**0.050**
								L-LTx	-1.62	0.105
								LA-LTx	-1.99	**0.046**
IL8	U	0.26	0.00	0.00	0.26	0.26	0.023	U-L	0.00	1.000
	L	0.26	0.00	0.00	0.26	0.26		U-LA	-2.09	**0.037**
	LA	3.83	0.21	0.12	3.50	3.90		U-LTx	-1.96	**0.050**
	LTx	3.36	0.21	0.15	3.21	3.50		L-LA	-2.09	**0.037**
								L-LTx	-1.96	**0.050**
								LA-LTx	-1.48	0.139
MCP1	U	0.11	0.00	0.00	0.11	0.11	0.392	U-L	0.00	1.000
	L	0.11	0.00	0.00	0.11	0.11		U-LA	-1.00	0.317
	LA	0.11	0.19	0.11	0.11	0.44		U-LTx	0.00	1.000
	LTx	0.11	0.00	0.00	0.11	0.11		L-LA	-1.00	0.317
								L-LTx	0.00	1.000
								LA-LTx	-1.00	0.317
SCGFb	U	7.82	0.00	0.00	7.82	7.82	0.013	U-L	0.00	1.000
	L	7.82	0.00	0.00	7.82	7.82		U-LA	-2.09	**0.037**
	LA	167.50	0.30	0.18	167.45	168.00		U-LTx	-2.24	**0.025**
	LTx	118.12	0.00	0.00	118.12	118.12		L-LA	-2.09	**0.037**
								L-LTx	-2.24	**0.025**
								LA-LTx	-2.09	**0.037**

SD: Standard deviation, **SE:** Standard error, **Min:** Minimum, **Max:** maximum.

P value was calculated using Independent-Samples Kruskal-Wallis Test. P values less than 0.05 was shown in bold. **U:** Untreated, **LA:** LPS/ATP (n=3)
